# Synthesis of Fe_2_SiO_4_-Fe_7_Co_3_ Nanocomposite Dispersed in the Mesoporous SBA-15: Application as Magnetically Separable Adsorbent

**DOI:** 10.3390/molecules25041016

**Published:** 2020-02-24

**Authors:** Monickarla Teixeira Pegado da Silva, Felipe Fernandes Barbosa, Marco Antonio Morales Torre, Jhonny Villarroel-Rocha, Karim Sapag, Sibele B. C. Pergher, Tiago Pinheiro Braga

**Affiliations:** 1 Laboratório de Peneiras Moleculares, Instituto de Química, Universidade Federal do Rio Grande do Norte, Natal 59078-970, RN, Brazil; monickarla@yahoo.com.br (M.T.P.d.S.); felipefbarboza@outlook.com (F.F.B.); 2 Departamento de Física, Universidade Federal do Rio Grande do Norte, Natal 59078-970, RN, Brazil; morales@dfte.ufrn.br; 3 Laboratorio de Sólidos Porosos, Universidad Nacional de San Luis, Instituto de Física Aplicada, 5700, San Luis D5700BPB, Argentina; jhoviro@gmail.com (J.V.-R.); ksapag@gmail.com (K.S.)

**Keywords:** magnetic nanocomposite, Fe_7_Co_3_ alloy, Fe_2_SiO_4_ oxide, adsorption, dyes

## Abstract

The mixture containing alloy and oxide with iron-based phases has shown interesting properties compared to the isolated species and the synergy between the phases has shown positive effect on dye adsorption. This paper describes the synthesis of Fe_2_SiO_4_-Fe_7_Co_3_-based nanocomposite dispersed in Santa Barbara Amorphous (SBA)-15 and its application in dye adsorption followed by magnetic separation. Thus, it was studied the variation of reduction temperature and amount of hydrogen used in synthesis and the effect of these parameters on the physicochemical properties of the iron and cobalt based oxide/alloy mixture, as well as the methylene blue adsorption capacity. The XRD and Mössbauer results, along with the temperature-programmed reduction (TPR) profiles, confirmed the formation of Fe_2_SiO_4_-Fe_7_Co_3_-based nanocomposites. Low-angle XRD, N_2_ isotherms, and TEM images show the formation of the SBA-15 based mesoporous support with a high surface area (640 m^2^/g). Adsorption tests confirmed that the material reduced at 700 °C using 2% of H_2_ presented the highest adsorption capacity (49 mg/g). The nanocomposites can be easily separated from the dispersion by applying an external magnetic field. The interaction between the dye and the nanocomposite occurs mainly by π-π interactions and the mixture of the Fe_2_SiO_4_ and Fe_7_Co_3_ leads to a synergistic effect, which favor the adsorption.

## 1. Introduction

Metal alloys and oxide nanocomposites containing Fe dispersed in ceramic matrices have been of great interest in the scientific community since these materials have a variety of applications [1,2] such as contrast enhancement in magnetic resonance images, gas sensors, magnetic fluids, magnetocaloric applications, magneto-optical data storage devices, magnetically separable adsorbents, among others [2,3,4,5]. The determining aspects in the physicochemical properties of these composites are related to the particle size distribution, morphology of particles, defects, purity of phases, and the fraction of atoms present on the surface compared to bulk atoms and the chemistry of the surface [2,6]. The main challenges are related to the synthesis of nanocomposites with defined shape, controlled composition, and adjustable interparticle distance [7].

It is known that alloys containing iron and cobalt represented by Fe_1-x_Co_x_ have high saturation magnetization and the physicochemical properties are influenced by their crystalline properties. The Fe_1-x_Co_x_ alloys, specifically the alloy designated as Fe_7_Co_3_, has shown very promising properties. This alloy has a body-centered cubic structure, high uniaxial magnetocrystalline anisotropy and high saturation magnetization [8,9,10,11]. Previous studies have indicated that the insertion of 30 wt% cobalt in relation to iron in the composition of the alloy provides a greater saturation magnetization at room temperature, leading to a wide application for Fe_7_Co_3_-based alloy such as microwave absorbing materials, ultra-high density magnetic recording medium, dye adsorption followed by magnetic separation, among others [5,11,12].

Other type of iron-based material is iron silicate (Fe_2_SiO_4_), which may have two distinct crystalline structures. The first is designated as α-Fe_2_SiO_4_, also known as fayalite with an olivine structure. The oxygen atoms are hexagonal close packing (hcp), the silicon is coordinated in tetrahedral positions and iron is located in two non-equivalent octahedral sites in equal proportions. On the other hand, the second type of iron silicate is named as γ-Fe_2_SiO_4_, it has a spinel structure. The oxygen atoms have a face-centered cubic (fcc) close packing, the silicon is located in tetrahedral sites and iron cations occupy equivalent octahedral positions. The fayalite is stable at room temperature, while the spinel phase is stable in a wide range of pressures and temperatures. Pure fayalite is paramagnetic at room temperature and becomes antiferromagnetic below 65 K [13,14,15]. Fayalite-based solids have been used in several applications such as refractory material, cement additive, ceramic pigment, and acid attack resistant containers as well as adsorption of cation contaminants. In addition, the Fe_2_SiO_4_ phase has excellent electrochemical properties and it is a more suitable anode compared to traditional cobalt orthosilicates for rechargeable lithium batteries [16,17,18].

Although oxide-based solids or alloys containing iron species, such as Fe_7_Co_3_ or Fe_2_SiO_4_ phases, have physicochemical properties and promising applications, the coupling of both phases in a composite is described as a solid with interesting characteristics compared to isolated phases. Investigations on this composite have confirmed the improvement of the physical and chemical properties of the final obtained material [19,20,21,22,23,24]. Previous studies have shown that the composite containing FeSiO_4_-Fe is an interesting material for application in catalysis. The addition of a zero valent Fe phase to the Fe_2_SiO_4_ generates desirable electrical and magnetic properties. The ability of the composite to self-heat up by magnetic induction helps to maintain the temperature at the level required for endothermal reactions such as catalytic cracking and steam or dry reform [25]. Although previous papers indicate the advantages of iron oxide-alloy composite synthesis [19,20,21,22,23,24,25], the synthesis of the FeSiO_4_-Fe_7_Co_3_ mixture remains a research theme to be explored, considering that no previous study have been published showing the properties of this composite.

The synthesis of nanocomposites containing alloy or oxide dispersed in a mesoporous silica has gain increased attention due to its high potential application in various research fields [26,27,28,29,30,31,32,33,34]. The iron-containing composites maintains the good properties of mesoporous silica matrix such as high surface area, adjustable pores size and volume, high chemical and mechanical resistance. The physicochemical properties of the iron phases are optimized when dispersed in a silica matrix, since silica acts as a dispersant agent of the iron phases [27,28]. Among the different mesoporous silicas, the Santa Barbara Amorphous (SBA)-15 has been greatly explored [29,30,31,32,33,34]. The SBA-15 silica (SBA—Santa Barbara Amorphous) has interesting textural properties, such as high specific area, uniform pore size distribution, wide wall thickness, small crystallite size, and regular mesoporosity [29,30,31,32]. The main advantages of SBA-15 as a dispersant agent of iron particles are related to its high surface-volume ratio, variable structure compositions, and high thermal stability, which optimize the physicochemical properties of the iron-based phases [33,34]. Although several studies confirm the feasibility of dispersing magnetic alloys and/or iron-based oxides in matrices containing mesoporous silica, the synthesis of the new Fe_2_SiO_4_-Fe_7_Co_3_-based composite dispersed in the mesoporous SBA-15 needs to be investigated in order to confirm its real viability, considering that no previous study was conducted for this type of nanocomposite.

In addition, it is worth mentioning that previous studies suggest that the temperature reduction and the hydrogen flow rate affect directly the physicochemical properties of the iron and cobalt based oxide-alloy mixture [35,36]. Therefore, different reduction temperatures and H_2_ flow rates were used in the synthesis of nanocomposite containing Fe_2_SiO_4_-Fe_7_Co_3_ dispersed in the mesoporous SBA-15 in order to evaluate these effects.

Among the different applications cited, for the materials based on Fe alloys and Fe-oxide dispersed in a mesoporous matrix, the adsorption of dyes present in wastewater followed by the magnetic separation has been greatly explored due to the fact that the contaminants can be easily removed from the water by applying an external magnetic field [5,37,38]. For example, Zhongli Wang et al. [5,37] synthesized the Fe_7_Co_3_ based nanocomposite dispersed in mesoporous carbon for adsorption of environmental contaminants and used a magnetic extraction method to remove the composite from the water. In another work, Chaoyang Jia et al. prepared a new magnetically separable adsorbent of iron oxide dispersed on silica with high adsorption capacity [38]. On the other hand, to the best of our knowledge, no study explored the potential of dye adsorption and magnetic separation for composite containing Fe_2_SiO_4_-Fe_7_Co_3_ dispersed in the mesoporous SBA-15. Thus, the present study shows the synthesis of alternative nanocomposite based on Fe_2_SiO_4_-Fe_7_Co_3_ supported in the SBA-15 mesoporous, it is presented the physicochemical characterization (structure, texture, morphology, and magnetic properties) and, finally, it was applied as magnetically separable adsorbent.

## 2. Results and Discussion

### 2.1. Structural Properties (X-ray Diffraction)

Based on the X-ray diffraction results, one can observe the influence of the reduction temperature and hydrogen content on the formation of the nanocomposite, since the intensities of the diffraction peaks varied depending on the temperature and the amount of H_2_ used in total flow rate.

**Figure 1:**
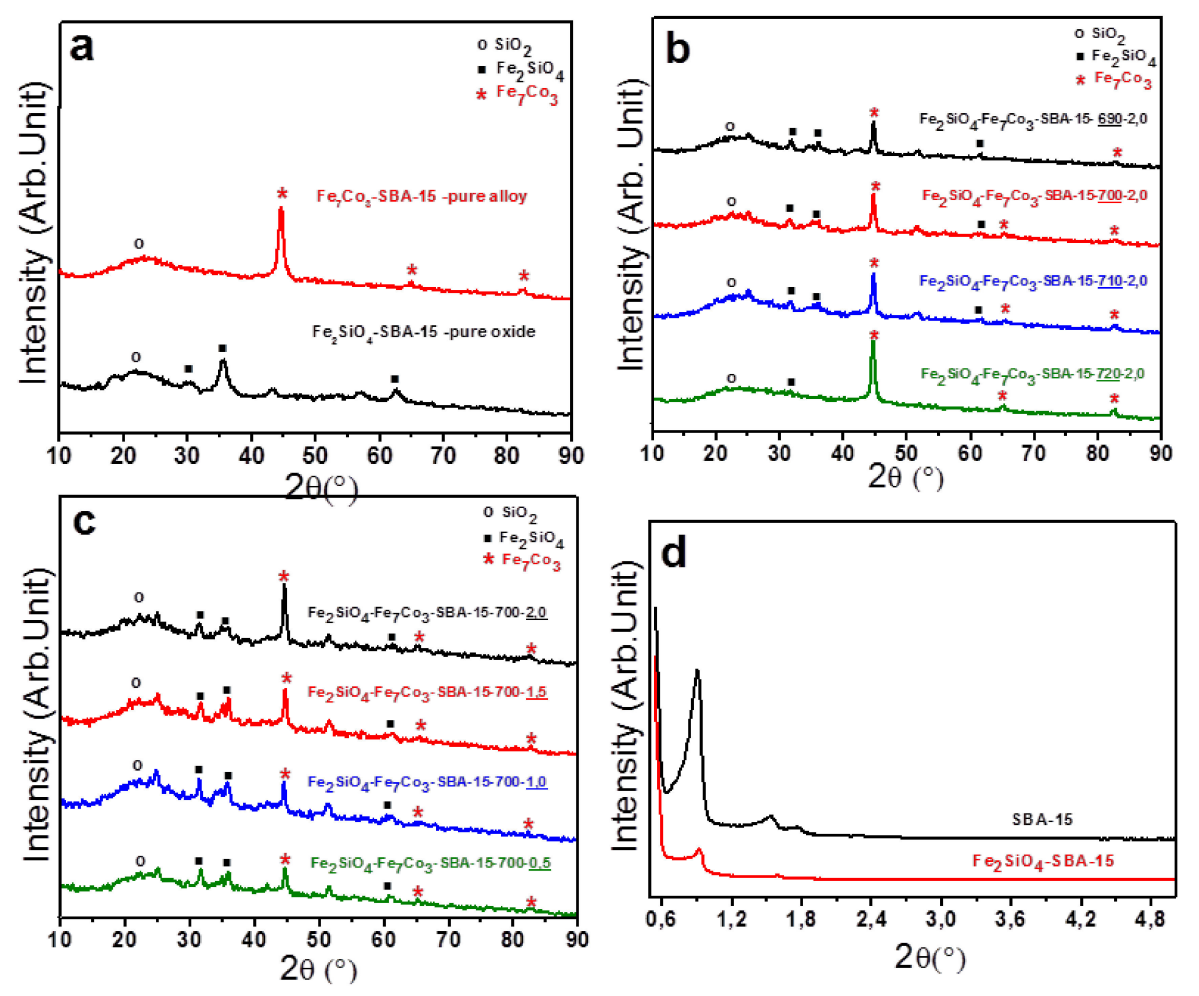
(**a**) Diffractograms of pure oxide (Fe_2_SiO_4_-Santa Barbara Amorphous (SBA)-15) and pure alloy (Co_7_Fe_3_-SBA-15); (**b**) Diffractograms concerning the influence of reduction temperature; (**c**) Diffractograms referring to the influence of H_2_ content; (**d**) Low-angle diffractograms for the support (SBA-15) before and after impregnation.

Initially, in Figure 1a, the characteristic diffractograms are presented for the samples containing oxide and pure alloy dispersed in the mesoporous SBA-15. It was also possible to observe, for both materials, the presence of a broad peak with 2θ values around 22°, characteristic of amorphous silica (SiO_2_) from the SBA-15 matrix. For pure oxide supported on SBA-15, well-defined peaks with 2θ values of approximately 30, 35, 62°, respectively, associated to iron silicate (Fe_2_SiO_4_), fayalite (JCPDS 00-071-1678), were visualized. In addition, for the pure oxide sample, cobalt ferrite formation (JCPDS 00-022-1086) was also observed. On the other hand, for the composite containing pure alloy supported on SBA-15, it was possible to identify three characteristic peaks concerning the Fe_7_Co_3_ phase (JCPDS 00-048-1816) with 2θ values in approximately 44.7, 65, and 82.5°, respectively. Thus, it confirms the total reduction of the oxide to form the alloy at 900 °C using pure H_2_. This is an expected result, since in this temperature range, iron and cobalt oxides are completely reduced [38,39]. The result of the Fe_7_Co_3_-SBA-15 solid indicates that the use of pure H_2_ was not adequate, since the use of high reducing gas content resulted in complete oxide reduction. Thus, it is necessary to use a mixture of H_2_ and N_2_ to reduce oxide only partially and lead to the formation of the dispersed oxide-alloy mixture supported in mesoporous silica.

The first study showing the effect of the reduction temperature on the formation of the oxide-alloy mixture, keeping the amount of hydrogen constant. The obtained results are presented in Figure 1b. This figure shows the diffractograms of samples starting from pure oxide (Fe_2_SiO_4_-SBA) submitted to different reduction temperature conditions with values ranging from 690 to 720 °C, maintaining the percentage reducing gas of 2% H_2_ and 98% N_2_ in the total flow rate of 25 mL/min.

One can observe that the increase in the reduction temperature leads to an increase in the peak intensity referring to Fe_7_Co_3_ alloy, which means that if the hydrogen amount is kept constant, the increase in the reduction temperature favors the reduction of oxide to alloy. Thus, it is possible to note that at 690 °C the sample Fe_2_SiO_4_- Fe_7_Co_3_-SBA-15-690-2.0 has a higher amount of oxide compared to other composites, while at 720 °C, the solid has a higher amount of alloy and only a small fraction of oxide.

In addition, the second study shows the effect of hydrogen concentration on the formation of composite, maintaining the reduction temperature at 700 °C constant, see Figure 1c. The diffractograms show the results concerning the four different H_2_ percentages. The effect of H_2_ content was less significant in composite formation compared to the influence of reduction temperature. However, it can be noted that the increase in the amount of H_2_ in the total flow rate leads to an increase in the amount of alloy in relation to oxide, confirming the role of hydrogen for reduction of oxide to alloy. Thus, an adequate control of the hydrogen amount in relation to nitrogen in the flow rate is fundamental to obtain the appropriate mixture of the Fe_2_SiO_4_ and Fe_7_Co_3_ phases dispersed in the silica matrix, taking into account that an excess of H_2_ will lead to a complete reduction of oxide.

Although all diffractograms qualitatively show two phases concerning Fe_7_Co_3_ alloy and Fe_2_SiO_4_-based oxide, the relative amount between phases changed depending on the reaction temperature and the amount of hydrogen applied, as can be seen in the peaks intensities. The structure refinement based on the Rietveld method was performed in order to quantify the observed phases and then calculate the crystallite size using the Scherrer equation. It was not possible to obtain qualitative data for the Fe_2_SiO_4_-SBA-15 sample due to the broad and low intensity peak for the Fe_2_SiO_4_-based oxide sample, and the diffractograms were quantified only for the oxide and alloy mixture. The results are presented in Table 1. The experimental and theoretical diffractograms obtained from the refinements are detailed in the supplementary materials (Figures S1 and S2).

It can be observed that the sample containing pure oxide was mainly composed by iron silicate and a small amount of cobalt ferrite, while the pure alloy corresponds to 100% of the Fe_7_Co_3_ phase, characterizing that the oxide was completely reduced to alloy. On the other hand, in Table 1, the refinement confirms that the increase in the reduction temperature and the increase in H_2_ content in total flow rate favor the reduction of oxide to alloy. The percentage of Fe_7_Co_3_ phase increased comparing the highest with the lowest reduction temperature and the highest with the lowest H_2_ content. This indicate that control of synthesis conditions is essential to obtain the desired percentage of alloy and oxide.

Furthermore, it was possible to observe that the crystallite size obtained from the diffractograms in Figure 1 did not vary significantly with the reduction temperature and with the amount of H_2_. This indicates that the increase from 690 to 720 °C was not significant to provide an evident increase in crystallite size due to particle sintering. The crystallites size values are between 14 and 20 nm for the different synthesized composites based on the data described in Table 1, confirming that the synthesis method used leads to the formation of small crystallites.

Previous papers describe the possible reactions that justify the formation of fayalite from iron oxides and silicon. A series of gas-solid reactions in the presence of H_2_ may be occurring, which leads to a series of redox reactions such as 2Fe^3+^ + 2Fe^0^ → 3Fe^2+^ and 2α-Fe^3+^ + 2Fe + 3SiO_2_ → 3α-Fe_2_SiO_4_ [40,41].

Low-angle XRD was performed in order to characterize the mesoporous structure. The results are presented in Figure 1d. The low angle diffractograms of the SBA-15 before and after impregnation of metals show three characteristic reflections with 2θ values of 0.9, 1.5, and 1.7° for planes (100), (110), and (200), respectively, which characterize a perfect hexagonal ordering of a typically mesoporous SBA-15. However, for the SBA-15 support after impregnation of iron and cobalt-based metals, there was a decrease in intensity and slight displacements in the characteristic reflections of the SBA-15 phase, which is due to a small disorder of the mesoporous structure. This may be related to the presence and dispersion of oxide in mesoporous silica, which will be confirmed below by N_2_ adsorption and desorption isotherms as well as by images obtained from transmission electron microscopy analysis.

### 2.2. Redox Properties of Pure Oxide (TPR-H_2_ Analysis)

**Figure 2:**
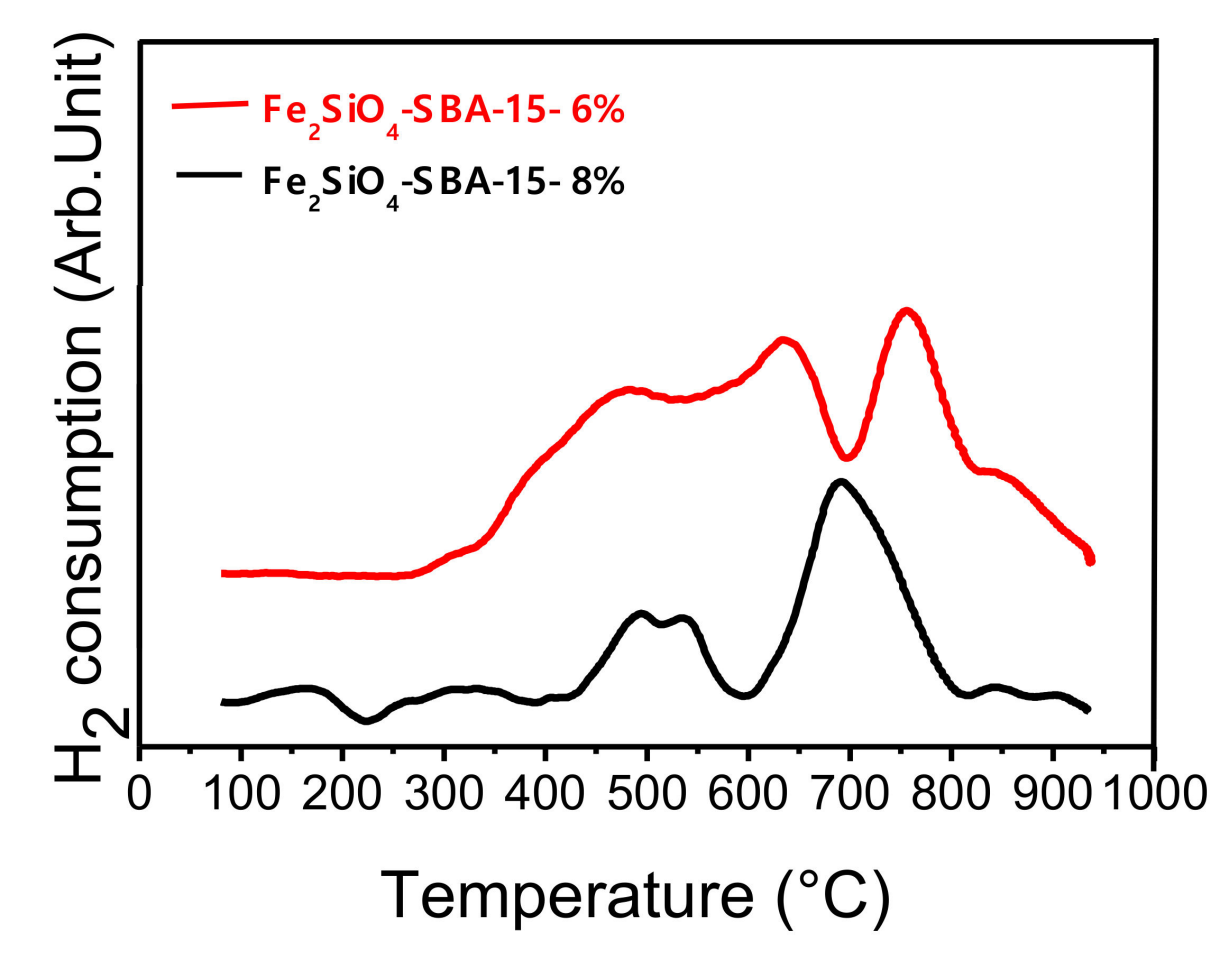
Temperature-programmed reduction (TPR) curves for the pure oxide dispersed on the SBA-15.

For the temperature-programmed reduction (TPR) analysis, the pure oxide sample (Fe_2_SiO_4_-SBA-15) was used with two different percentages of hydrogen in the reducing mixture. The analysis was performed to evaluate the reduction temperature of oxide to alloy and the effect of H_2_ content. The percentages containing 6 and 8% of H_2_ in N_2_ were used in the total flow rate of 25 mL/min. The TPR profiles are presented in Figure 2. According to the literature, solids containing iron and cobalt dispersed in silica begin to reduce at temperature between 250 and 300 °C. It usually has two or three peaks related to the reduction of iron and cobalt oxides concerning the phases of cobalt ferrite and fayalite dispersed in the silica matrix as observed in the diffractions of Figure 1 [41,42,43,44,45]. The TPR curves show two distinct reduction stages, the first between 350 and 650 °C and the second between 650 and 850 °C, approximately. These peaks were related to the cobalt oxide (Co^2+^→ Co^+^ →Co^0^) and iron oxide reduction (Fe^3+^→ Fe^2+^→ Fe^0^) present in the Fe_2_SiO_4_ and CoFe_2_O_4_ phases identified in the Fe_2_SiO_4_-SBA-15 sample before reduction (Figure 1a).

The first reduction range can be attributed to the first stage of iron oxide (III) reduction as well as the reduction of Co^2+^, both present in the iron and cobalt oxides structure. The second peak can be related to the second stage of iron reduction, Fe^2+^ to Fe^0^, leading to Fe_7_Co_3_ alloy formation, visualized in diffractograms which iron and cobalt are completely reduced.

The interaction of iron and cobalt oxides in the structure of the silica matrix leads to a change in the structure of iron oxide phases, justifying the formation of the Fe_2_SiO_4_ phase [39]. The second range referring to the reduction of Fe^2+^ to Fe^0^ may be related to the reduction of iron silicate. It is worth to highlight that the iron silicate and cobalt ferrite phases, observed in the XRD results before reduction, are very resistant structures to be reduced and, therefore, the complete reduction of these phases may not have been completely observed in the temperature range studied [41,45]. It is important to mention that metal oxides particles located internally in the pores are more difficult to reduce compared to particles positioned on the outer surface [46,47]. Thus, the reduction range at higher temperatures may also be related to particles of Fe and Co oxides located within the pores of the SBA-15.

TPR profiles can be correlated with diffractograms, since the effect of the reduction temperature on the percentage of alloy in relation to oxide was observed, Figure 1b and Table 1. It is possible to observe the second reduction range related to the partial reduction of Fe^2+^ and Co^2+^ to Fe^0^ and Co^0^ at the temperature range chosen between 690 and 720 °C, where the oxide and alloy mixture was observed in the diffractograms, leading to the formation of a fraction of the Fe_7_Co_3_ alloy. The increase in the alloy content in relation to oxide observed in the XRD results can also be seen in the TPR profiles, once that at 720 °C the consumption of H_2_ is higher compared to the temperature of 690 °C, indicating a higher reduction rate with the increase in temperature.

The second effect studied concerning the influence of the H_2_ percentage on the ability to reduce oxide to alloy can also be seen in TPR results. In the XRD data, it has already been shown that the increase in hydrogen content intensifies the reduction of oxide to alloy. This phenomenon can be seen clearly in TPR profiles, considering that when higher H_2_ content was used the reduction occur at lower temperatures, confirming the agreement between the two techniques. For example, the TPR performed with 6% of H_2_ the total reduction occurred at approximately 780 °C, while using 8% of hydrogen the complete reduction occurred at lower temperatures around 700 °C.

### 2.3. Chemical State Surface of Iron, Cobalt and Silicon (XPS Analysis) 

The samples Fe_2_SiO_4_-SBA-15, Fe_2_SiO_4_-Fe_7_Co_3_-SBA-15-700-0.5, and Fe_2_SiO_4_-Fe_7_Co_3_-SBA-15-700-1.0 were selected for XPS analysis to evaluate the chemical state surface of the composite observed in the XRD results and compare them with the two pure phases. The results are present in Figure S5. The wide-scan XPS spectrum with binding energies from 0 to 800 eV for the Fe_2_SiO_4_-SBA-15 solid showed four characteristic peaks with binding energy values of 103.4, 532, 712, and 781 eV related to Si2p, O1s, Fe2p, and Co2p, respectively. The binding energy at 532 eV refers to the typical O1s of SiO4^−2^ present in the Fe_2_SiO_4_ phase and the Si2p is related to silicon oxide from the SBA-15 matrix. In addition, the spectra for Fe_2_SiO_4_-Fe_7_Co_3_-SBA-15-700-0.5 and Fe_2_SiO_4_-Fe_7_Co_3-_SBA-15-700-1.0 solids showed peaks with binding energies of approximately 103.4, 532, 710.1, and 781.1 eV attributed to Si2p, O1s, Fe2p, and Co2p, respectively. The Co^2+^, Fe^3+^/Fe^2+^ and Fe^0^ species are related to peaks at 781.1, 712 eV and 710.1, respectively, as presented in Figure S5. The percentage of elements from the full XPS spectrum are shown in Table S1, indicating the quantity of each element on the surface. The observed binding energy values corroborate with other previously published papers [48,49,50]. The XPS spectra are in accordance with the diffractograms, see Figure 1, considering that binding energy of Fe^2+^ and Fe^0^ from the Fe_7_Co_3_ and Fe_2_SiO_4_ phases, as already indicated in the XRD results.

The XPS results indicate that the impregnation method was successfully performed, since the structure of Fe_2_SiO_4_/SBA-15 solid and the composite Fe_2_SiO_4_-Fe_7_Co_3_-SBA-15 are present on the solid surface, which is a fundamental aspect for application in the adsorption of organic molecules.

### 2.4. Chemical Environment of the Iron (Mössbauer Spectroscopy)

The spectrum at 300 K for the Fe_2_SiO_4_-SBA-15 solid is fit with two components, a sextet due to a slow relaxing Fe moments in the Fe oxide phase and a doublet due to a fast relaxation Fe moments present in Fe oxide superparamagnetic nanoparticles (SPM), Figure S4a. A large fraction of the Fe-oxide phase is in the superparamagnetic regime. The B_hf_ obtained for the slow relaxing Fe moments is smaller than the thermally blocked bulk-CoFe_2_O_4_ particles [51], indicating that the cobalt and iron spin moments are experiencing a slow relaxation and, therefore, diminishing the hyperfine magnetic field.

The Mössbauer spectrum for sample Fe_7_Co_3_-SBA-15 has two components, a magnetic and a paramagnetic, Figure S4b. The magnetic component is ascribed to the Fe_7_Co_3_ alloy, it has a hyperfine magnetic field (B_hf_) and isomer shift (IS) typical for the metal alloy. In order to find the sextet that best fit the spectrum, it was performed a fitting using several sextets with a distribution of B_hf_ and a distribution of IS. The distribution shows a peak at *B_hf_* = 35.7 T at an IS of 0.032 mm/s. These results indicate that those parameters are adequate to fit the component ascribed to the alloy. The paramagnetic component (SPM) has a small Mössbauer spectral contribution and IS similar to the Fe_7_Co_3_ phase, this component is due to a very small metal nanoparticles in the superparamagnetic regime.

Mössbauer results for the solid Fe_2_SiO_4_-Fe_7_Co_3_-SBA-15-700-2.0 is presented in Figure S4c. The spectrum at 300 K is deconvoluted using three components, two paramagnetic and one magnetic subspectra. The component that has the largest relative area is the sextet and is ascribed to the Fe_7_Co_3_ alloy. It shows the nuclear transition between excited and ground states of magnetically split nuclear moment levels. Each peak is regarded to the Zeeman Effect and has an energy of *E* = −Δm. B_hf_ where Δm is one of the allowed transition between the nuclear levels from the excited (*I* = ±3/2) and the ground (*I* = ±1/2) states, and B_hf_ is the hyperfine magnetic field. The magnetic field is provided by a non zero density of states at the Fermi level due to electrons in Fe and Co atoms. The *B_hf_* = 35.8 T of the present sample is very close to the B_hf_ of Fe_7_Co_3_ [52]. Usually, the Mössbauer spectrum of pure Fe_7_Co_3_ alloy is fit using several sextets with B_hf_ ranging from 31.4 T to 36.9 T, and the sextets with the largest (>40%) absorption areas are the sextets with the largest B_hf_. Herein, besides the sextet there are two paramagnetic components. These components are doublets related to Fe_2_SiO_4_ and Fe^3+^-silicate phases. The bulk Fe_2_SiO_4_ has a Fe^2+^ charge state and is antiferromagnetic with a Néel transition at 64.5 K [53], thus, at 300 K this phase is in the paramagnetic regime. The Fe^2+^ occupies two non-equivalent octahedral sites in equal proportions. From the literature, a bulk sample has an IS ranging from 0.99–1.02 mm/s and QS ranging from 2.91–3.02 mm/s, while nanostructured samples have similar IS values and reduced QS values [54]. In the present results (Table S2), it was considered a representative single doublet to fit the Fe_2_SiO_4_ phase, where the large IS and QS are typical of Fe^2+^. On the other hand, the subspectrum for the Fe^3+^-silicate is paramagnetic at 300 K and its spectrum has hyperfine parameters similar to microwaved silica coated maghemite sample [55]. Thus, Mössbauer results are in agreement with the obtained diffractograms for the nanocomposites, since in both analyses, the presence of Fe_7_Co_3_ and Fe_2_SiO_4_ phases were observed.

### 2.5. N_2_ Adsorption and Desorption Isotherms (Textural Properties)

**Figure 3:**
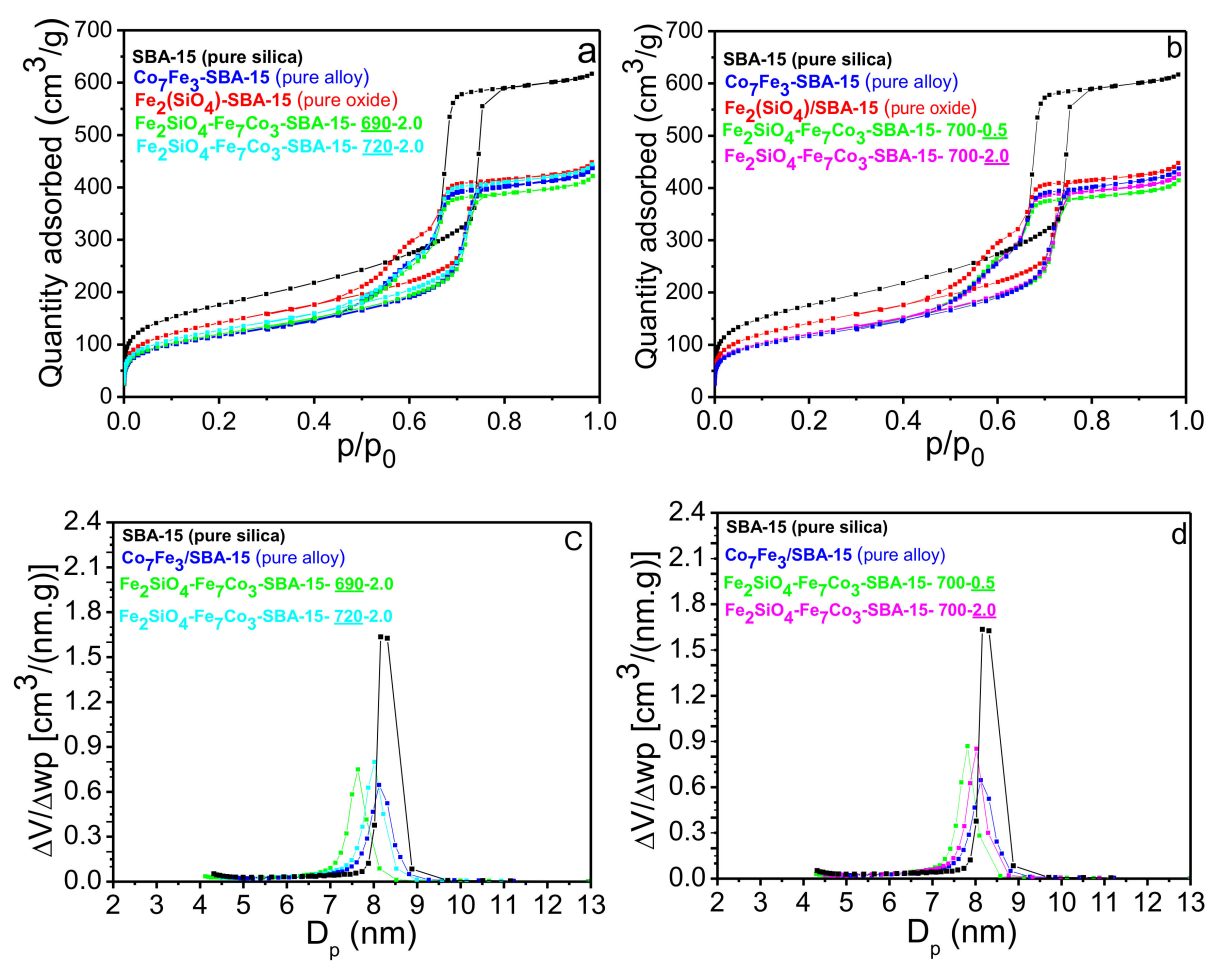
(**a**) N_2_ isotherms for pure SBA-15, Fe_2_SiO_4_-SBA-15, Co_7_Fe_3_-SBA-15, and the two extremes of reduction temperature studied; (**b**) N_2_ isotherms for the pure SBA-15, Fe_2_SiO_4_-SBA-15, Co_7_Fe_3_-SBA-15, and the two extremes of hydrogen quantity studied; (**c**) and (**d**) Pore diameter distribution for the different materials described in Figure 3a,b.

Figure 3a displays the N_2_ adsorption and desorption isotherms of the reduced materials at 690 and 720 °C (two extremes of temperature) keeping the H_2_ content constant as well as for oxide, alloy, and pure SBA-15. It was observed that both materials containing pure silica and impregnated silica present regular mesoporosity and type IV isotherms, typical of mesoporous materials, with type H1 hysteresis for pure silica (SBA-15) and type H2 (b) for other impregnated materials. H1 hysteresis are characteristics of cylindrical mesoporous materials and uniformly distributed typical of silica (SBA-15, MCM-41, KIT-6). The H2 (b) hysteresis type are considered an intermediate of the anterior hysteresis, characteristic of materials with complex porous structure and is associated with partially blocked pores and present an interconnected pores networks with different sizes and shapes. Therefore, this blocking effects may be associated with partial pore filling by the Fe_7_Co_3_ and/or Fe_2_SiO_4_ phases due to the impregnation methodology used.

No significant change in textural properties before and after impregnation of iron and cobalt oxides as well as after reduction in the different temperatures were observed, indicating that the different stages of oxidation and reduction in the different temperatures did not affect the isotherm type formed. On the other hand, the hysteresis type and amount of N_2_ adsorbed changed significantly by comparing pure silica with the other composites that were impregnated with Fe and Co. The textural properties extracted from the N_2_ physisorption isotherms are described in Table 2. Before impregnation of Fe_2_SiO_4_ oxide, the pure SBA-15 material has a specific surface area of 640 m^2^/g. However, after impregnation, the specific surface area values, as well as the total pore volume decreased as can be observed in Table 2. According to the literature [56], the oxide crystallites size and their reduction depend heavily on the pore diameter of the silica. Thus, the partial introduction of oxides in mesoporous silica promote a decrease in the specific surface area and total pore volume. For example, specific area values decreased from 640 to 510 m^2^/g and total pore volume from 0.94 to 0.69 cm^3^/g comparing pure silica with Fe_2_SiO_4_-SBA-15 solid. This confirms that the changes in the specific surface area were not too drastic and that the good textural characteristics of the silica matrix were maintained in the nanocomposite, considering that it is a fundamental aspect to provide high dispersion of the Fe_7_Co_3_ and Fe_2_SiO_4_ phases in the mesoporous matrix. After reduction at different temperatures, there is also a slight decrease in the surface area and pore volume. However, comparing the textural properties of the different temperatures used, it is perceived that temperature variation did not significantly affect the values of surface area, pore volume, and pore diameter.

The change in the hysteresis type of the isotherms due to the variation in the pore structure for the SBA-15 is in accordance with the low angle diffractions (Figure 1d), which showed a slight change in peaks after the impregnation stage. Despite the alteration of textural properties after impregnation, the materials reduced in the different temperatures showed isotherms with very similar profile, indicating that the pore structure was not significantly affected with the increase in the reduction temperature. These results are in accordance with the crystallites size calculated from the diffractograms of Figure 1b, since the crystallites size, Table 1, was also not significantly affected with the reduction temperature variation.

In addition, the effect of H_2_ content used for nanocomposite synthesis on the textural properties of different solids was also studied. The results of N_2_ adsorption-desorption isotherms for the amounts of 0.5% and 2.0% of H_2_ studied (two extremes of H_2_ content) are present in Figure 3b. It can be observed that the results of the textural properties obtained with the variation of H_2_ flow rate were very similar to the study of temperature variation (Figure 3a). The samples presented the same type of isotherm and hysteresis as well as the specific surface area values, volume and pore diameter were practically the same. These results suggest that metals inserted by impregnation are mainly on the catalyst surface, as presented in the XPS results (Figure S2), and a small fraction of pores is partially blocked by oxide and oxide-alloy mixture (Fe_2_SiO_4_ and the Fe_2_SiO_4_-Fe_7_Co_3_ mixture). Despite the small change in textural properties after impregnation, the mesoporous structure of the SBA-15 support was practically maintained, corroborating with the low angle diffractions (Figure 1d). The variation in the percentage of H_2_ also did not significantly affect the crystallite size, Table 1, corroborating with the texture properties values presented in Table 2.

Figure 3c,d present the pore diameter distribution curves for pure SBA-15, Fe_2_SiO_4_-SBA-15, and Fe_2_SiO_4_-Fe_7_Co_3_-SBA-15 with their respective variations in reduction temperature and H_2_ flow rate. A homogeneous distribution was observed in the mesopores range for the different samples. It can be observed that impregnated materials have an average pore size smaller than pure SBA-15 due to partial pore blocking by oxide and alloy as previously described.

### 2.6. Morphological Properties (SEM-FEG and TEM)

High resolution scanning electron microscopy analyses were performed to obtain information related to the morphology of synthesized nanocomposites as well as confirm the maintenance of silica matrix morphology before and after impregnation of oxide and/or alloy as well as to observe the particle size distribution after impregnation and reduction through the construction of histograms.

**Figure 4:**
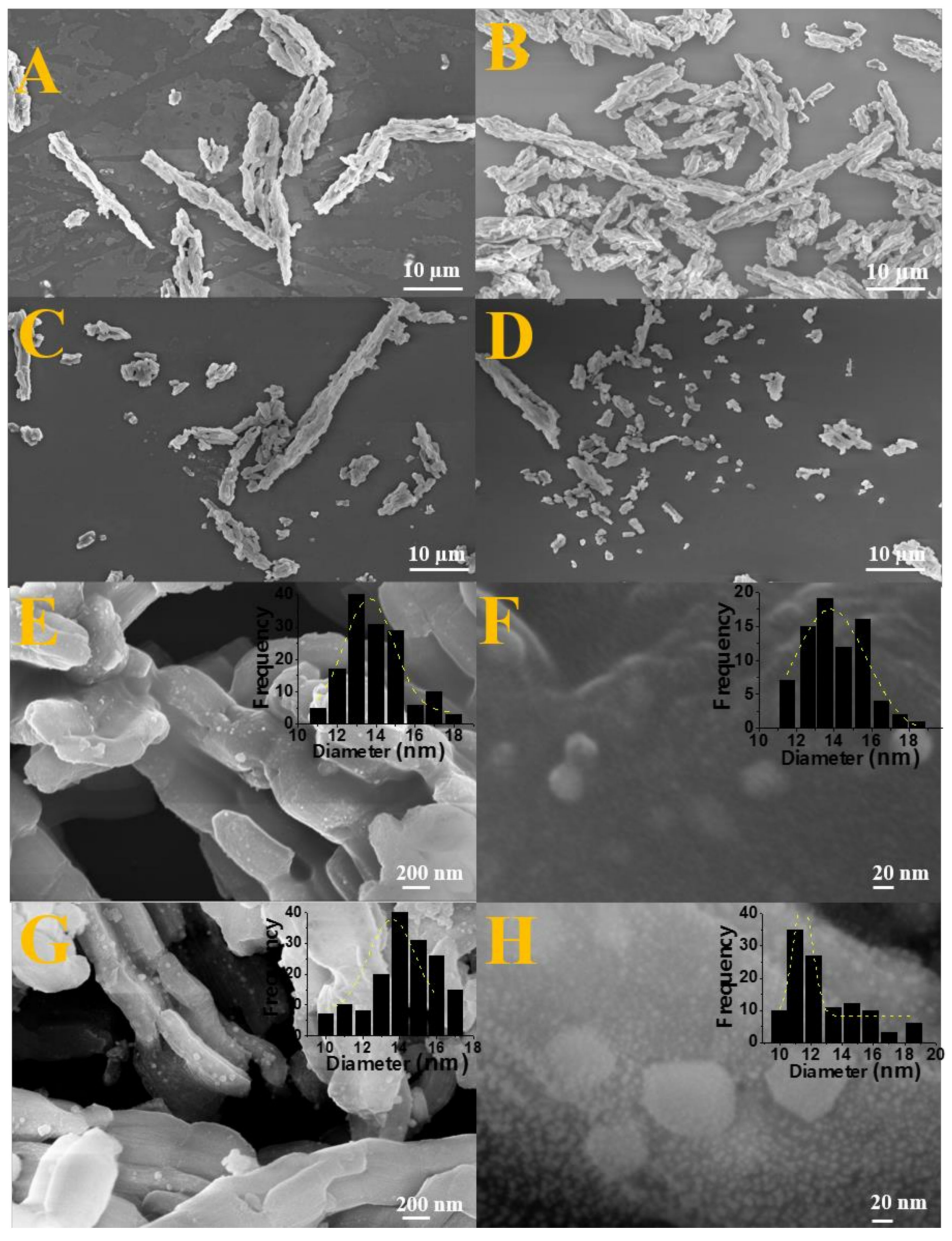
(**A**) Micrographs of the pure SBA-15; (**B**–**D**) Images after impregnation on SBA-15 support. (**E**,**F**) Micrographs with the particle size distribution for sample Fe_2_SiO_4_-Co_7_Fe_3_-SBA-15-700-2.0. (**G**,**H**) Image with the particle size distribution for solid Fe_2_SiO_4_-Co_7_Fe_3_-SBA-15-700-0.5.

In Figure 4A, associated to pure silica before impregnation, it is possible to observe a highly ordered matrix with uniform hexagonal rod-like morphology and longitudinally arranged characteristic of the SBA-15 silica [47,56]. It is possible to notice that the typical morphology of SBA-15 is maintained in solids after impregnation and calcination for oxide formation as well as after the reduction stage for nanocomposite formation, since the rods were preserved comparing the images of Figure 4A–D. These results corroborate with the low angle diffractograms (Figure 1d) that showed the maintenance of the pore structure after impregnation and they are also in accordance with the N_2_ adsorption-desorption isotherms that indicated the maintenance of the typical mesoporosity for the SBA-15 after the Fe and Co insertion (Figure 3).

Furthermore, in order to complement the FEG-SEM analyses, particle size distribution was obtained for Fe_2_SiO_4_-Fe_7_Co_3_-SBA-15-700-0.5 and Fe_2_SiO_4_-Fe_7_Co_3_-SBA-15-700-2.0 solids from the images present in Figure 4E–H. In addition, an elementary analysis was carried out by EDS and the information is presented in Table S3, confirming the presence of Fe, Co, Si, and O. It was observed that the sample reduced using 2.0% of H_2_ has particle sizes ranging from 11 to 18 nm with an average size of 14 nm. However, the nanocomposite prepared with 0.5% of hydrogen in the reducing mixture showed particle sizes ranging from 10 to 19 nm with an average value of 16 nm according to histograms inserted in Figure 4. The particle size values observed by SEM were in accordance with the crystallites size calculated from the diffractograms using the Scherrer equation (Table 1) and similarly the variation of the H_2_ percentage used in the synthesis did not significantly affect the particles size, considering that both samples presented average particle size very close. This result shows that there is not aggregation of nanoparticles.

**Figure 5:**
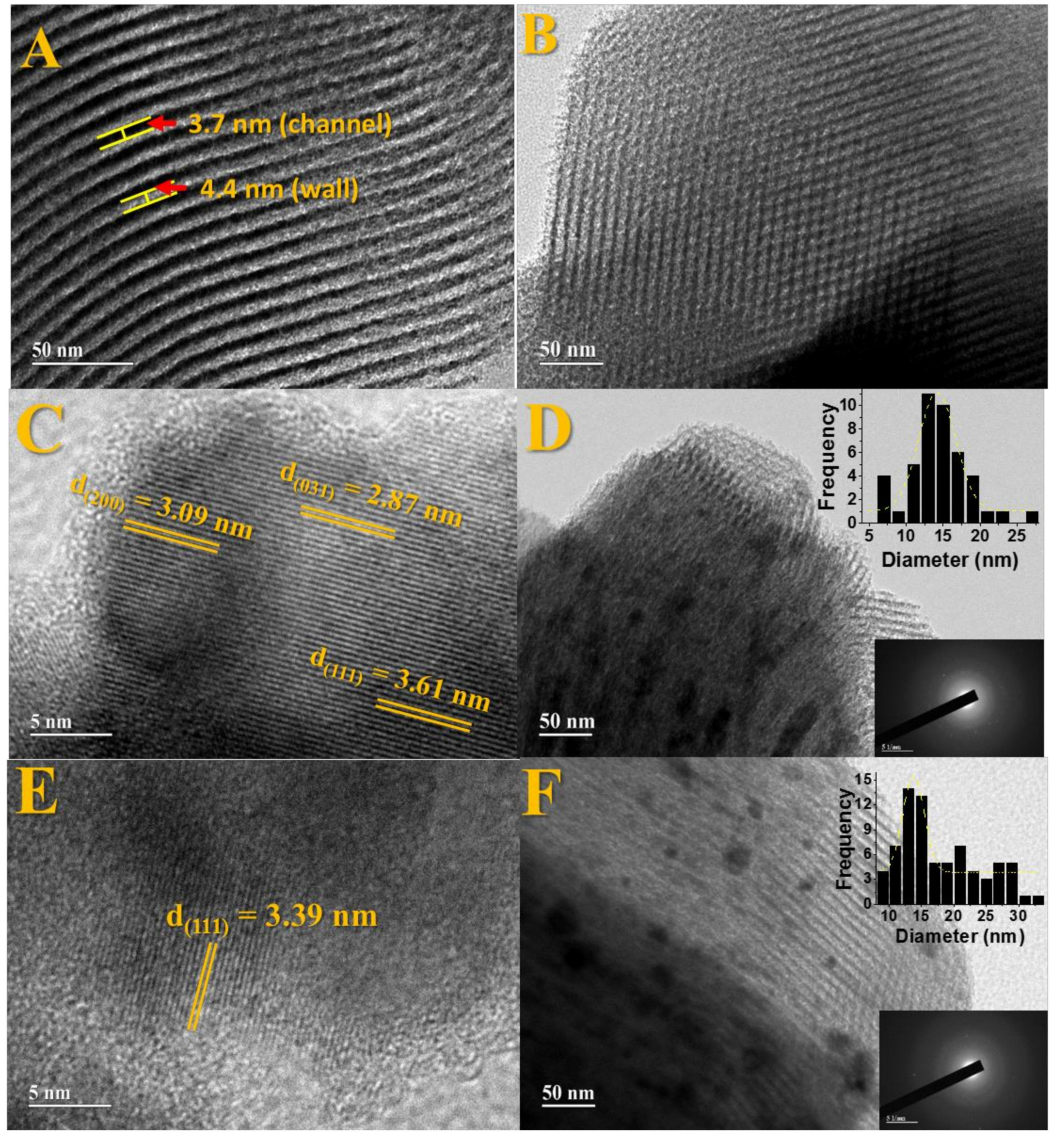
TEM images for solids: (**A**,**B**) pure SBA-15, (**C**,**D**) for the solid Fe_2_SiO_4_-Co_7_Fe_3_-SBA-15-700-2.0, and (**E**,**F**) for the sample Fe_2_SiO_4_-Co_7_Fe_3_-SBA-15-700-0.5 using different approximations. The figures inserted above are related to particle size distribution and below concerning the selected area electron diffraction (SAED).

Transmission electron microscopy (TEM) was performed for pure SBA-15, Fe_2_SiO_4_-Fe_7_Co_3_-SBA-15-700-0.5, and Fe_2_SiO_4_-Fe_7_Co_3_-SBA-15-700-2.0 solids in order to confirm the mesoporous structure of these solids, to visualize the partial confinement of the nanocomposite and determine the particle size distribution. The different images obtained are presented in Figure 5. One can observe that the ordered arrangement of pores consists of long mesochannels, which once again agree with low angle XRD diffractograms (Figure 1d). Figure 5B shows a 2D two-dimensional hexagonal arrangement of uniformly sized cylindrical mesoporous channels characteristic of the SBA-15 [57]. For pure SBA-15, Figure 5A, the wall thickness and the channel distance (pores) were calculated, obtaining an interpore distance of 3.7 nm and wall thickness of 4.4 nm. From the low angle diffractograms and the values obtained in the N_2_ isotherms, it was possible to determine the wall thickness for the SBA-15 and compare with the value obtained by TEM. The values obtained are described in Table S4. There is an agreement in the data obtained by the two techniques.

The images presented in Figure 5C–F show well-defined lattice fringes related to mesoporous SBA-15 partially filled by the Fe_7_Co_3_ and Fe_2_SiO_4_ phases, which are also in accordance with low-angle XRD diffractograms (Figure 1d) and N_2_ isotherms (Figure 3).

**Figure 6:**
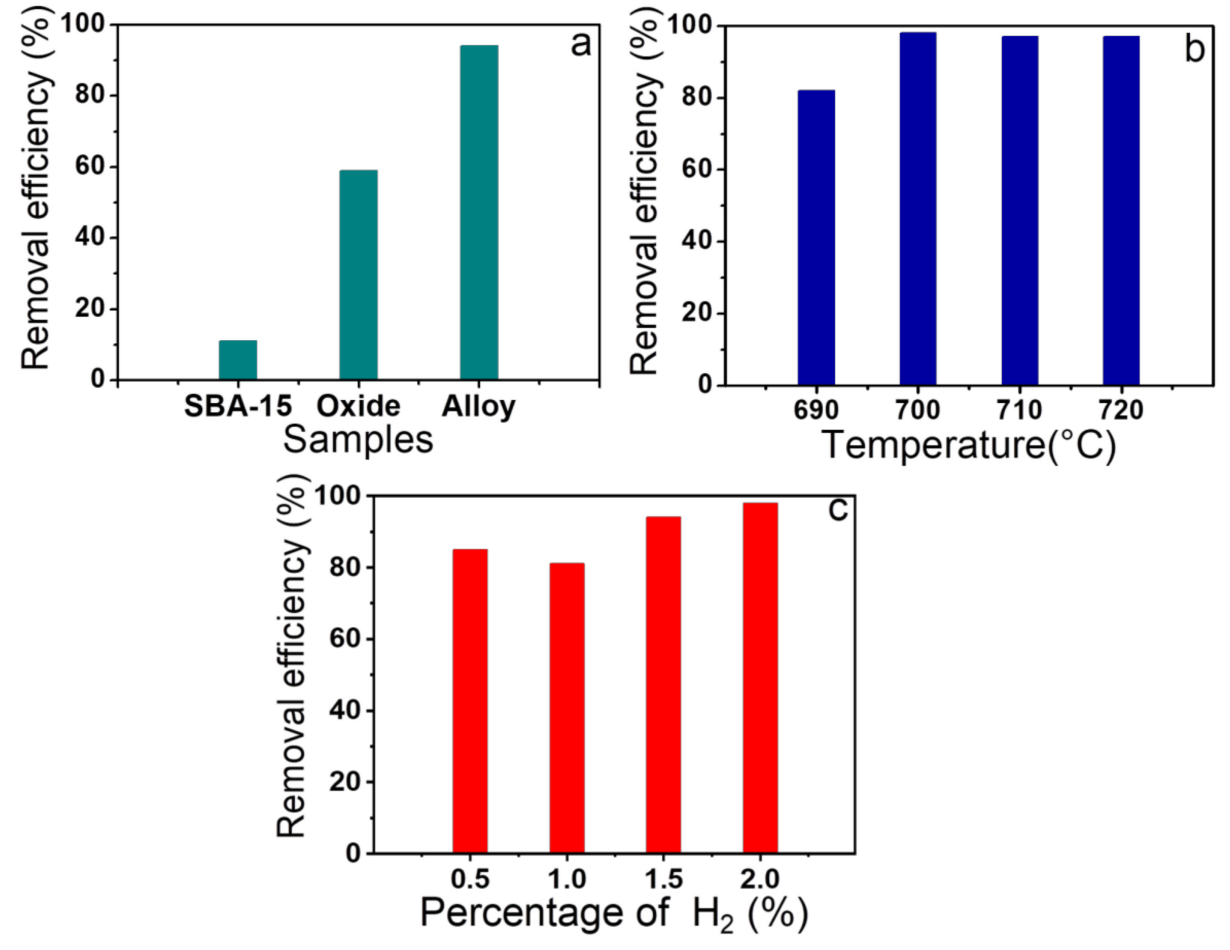
(**a**) Dye removal efficiency for pure SBA-15, oxide, and alloy dispersed on silica matrix; (**b**) Removal efficiency for the study of the reduction temperature variation; (**c**) Removal efficiency concerning the study of the effect of H_2_ content.

Figure 5C,D refers to the Fe_2_SiO_4_-Fe_7_Co_3_-SBA-15-700-2.0 solid, and Figure 5E,F are related to the Fe_2_SiO_4_-Fe_7_Co_3_-SBA-15-700-0.5 material. Image 5C shows the interplanar distances of 2.87 nm, 3.09 nm, and 3.61 nm, respectively, for the planes (031), (200), and (111) referring to the solid Fe_2_SiO_4_-Fe_7_Co_3_-SBA-15-700-2.0, which is in accordance with the pattern shown in the diffractograms of Figure 1. Figure 6E also displays a distance of 3.39 nm, referring to the plane (111) for Fe_2_SiO_4_-Fe_7_Co_3_-SBA-15-700-0.5 and also corroborates with the JCPDS 00-071-1678 card.

Moreover, Figure 5D,F present a histogram with particle size distribution. An average particle size around 17.8 nm was observed for the Fe_2_SiO_4_-Fe_7_Co_3_-SBA-15-700-2.0 composite and 14.4 nm for the Fe_2_SiO_4_-Fe_7_Co_3_-SBA-15-700-0.50 sample. The particle sizes values determined by TEM corroborate with the values obtained by the SEM images and also the crystallite size extracted from diffractograms using the Scherrer equation. The images inserted at the bottom of Figure 5D,F, referring to selected area electron diffraction, confirm the polycrystalline nature of the nanoparticles related to the Fe_7_Co_3_ and Fe_2_SiO_4_ phases observed in XRD and Mössbauer results.

### 2.7. Adsorption Tests

Initially, in order to investigate the adsorption capacity of magnetic nanocomposite using three types of dyes, the Fe_2_SiO_4_-Fe_7_Co_3_-SBA-15-700-2.0 sample was selected. The results are presented in Figure S5. Thus, it was possible to observe the performance of the material Fe_2_SiO_4_-Fe_7_Co_3_-SBA-15-700-2.0 for the adsorption of three dyes, with an adsorption capacity of 7 mg/g for methyl orange, 27 mg/g for rhodamine B, and 49 mg/g for methylene blue. Therefore, as methylene blue presented the best adsorption results for the selected solid, all samples described in Table S5 were tested to evaluate which material has the highest adsorption capacity among the synthesized solids. In addition, a study was also conducted for the pure SBA-15, Fe_2_SiO_4_-SBA-15, and Fe_7_Co_3_-SBA-15 in order to investigate the adsorption capacity of the Fe_7_Co_3_ and Fe_2_SiO_4_ phases separately and confirm the positive effect of oxide and alloy mixture on the dye adsorption efficiency. The obtained results are presented in Figure 6. In Figure 6a, one can observe that the pure SBA-15 silica matrix has low adsorption capacity compared to other solids after oxide impregnation and alloy formation, confirming that the presence of Fe and Co is fundamental to obtain better results for dye removal.

The adsorption capacities of magnetic nanocomposites for samples varying the reduction temperature used in the synthesis increases significantly with the temperature increase from 690 °C (41 mg/g) to 700 °C (49 mg/g) and remains virtually constant, with the temperatures between 710 and 720 °C (48 mg/g), Figure 6b. These results indicate that the presence of a greater amount of alloy in relation to oxide is necessary to obtain better adsorption results, whereas the Fe_2_SiO_4_-Fe_7_Co_3_-SBA-15-690-0.5 sample has lower amount of alloy compared to the Fe_2_SiO_4_-Fe_7_Co_3_-SBA-15-700-2.0 material, justifying its best performance. However, the reduction temperature above 700 °C, despite increasing the alloy amount in relation to oxide, does not affect the adsorption capacity, considering that the percentage degradation was quite similar for the solids reduced at temperatures of 700, 710, and 720 °C, indicating that from a certain amount of the alloy in relation to oxide the adsorption performance is not significantly affected.

In addition, it was also studied the influence of the amount of hydrogen used in the synthesis on adsorption performance, Figure 6c. Similar to the effect of the reduction temperature, adsorption capacity increases with the increase in the amount of alloy in relation to oxide, considering that the different solids presented the following results in ascending order Fe_2_SiO_4_-Fe_7_Co_3_-SBA-15-700-1.0 (40 mg/g), Fe_2_SiO_4_-Fe_7_Co_3_-SBA-15-700-0.5 (43 mg/g), Fe_2_SiO_4_-Fe_7_Co_3_-SBA-15-700-1.5 (47 mg/g), and Fe_2_SiO_4_-Fe_7_Co_3_-SBA-15-700-2.0 (49 mg/g). Thus, the higher adsorption capacity of the sample Fe_2_SiO_4_-Fe_7_Co_3_-SBA-15-700-2.0 (oxide and alloy mixture) compared to Fe_7_Co_3_-SBA-15 (isolated pure alloy) and Fe_2_SiO_4_-SBA-15 (isolated pure oxide) samples confirm the best nanocomposite properties containing the Fe_7_Co_3_ and Fe_2_SiO_4_ phase mixture in relation to the same isolated phases. Furthermore, it was observed that control of synthesis conditions such as reduction temperature and H_2_ content also affect adsorption capacity, considering that the alloy amounts in relation to oxide in the mixture affect the physicochemical properties of nanocomposites as shown in previous characterizations and consequently affect the adsorption performance of methylene blue. These results suggest that there is a synergy between the Fe_7_Co_3_ and Fe_2_SiO_4_ phases that positively affects dye adsorption. Previous studies have reported a synergy between alloy and oxide phases that positively affect the interaction of organic molecules [58].

**Figure 7:**
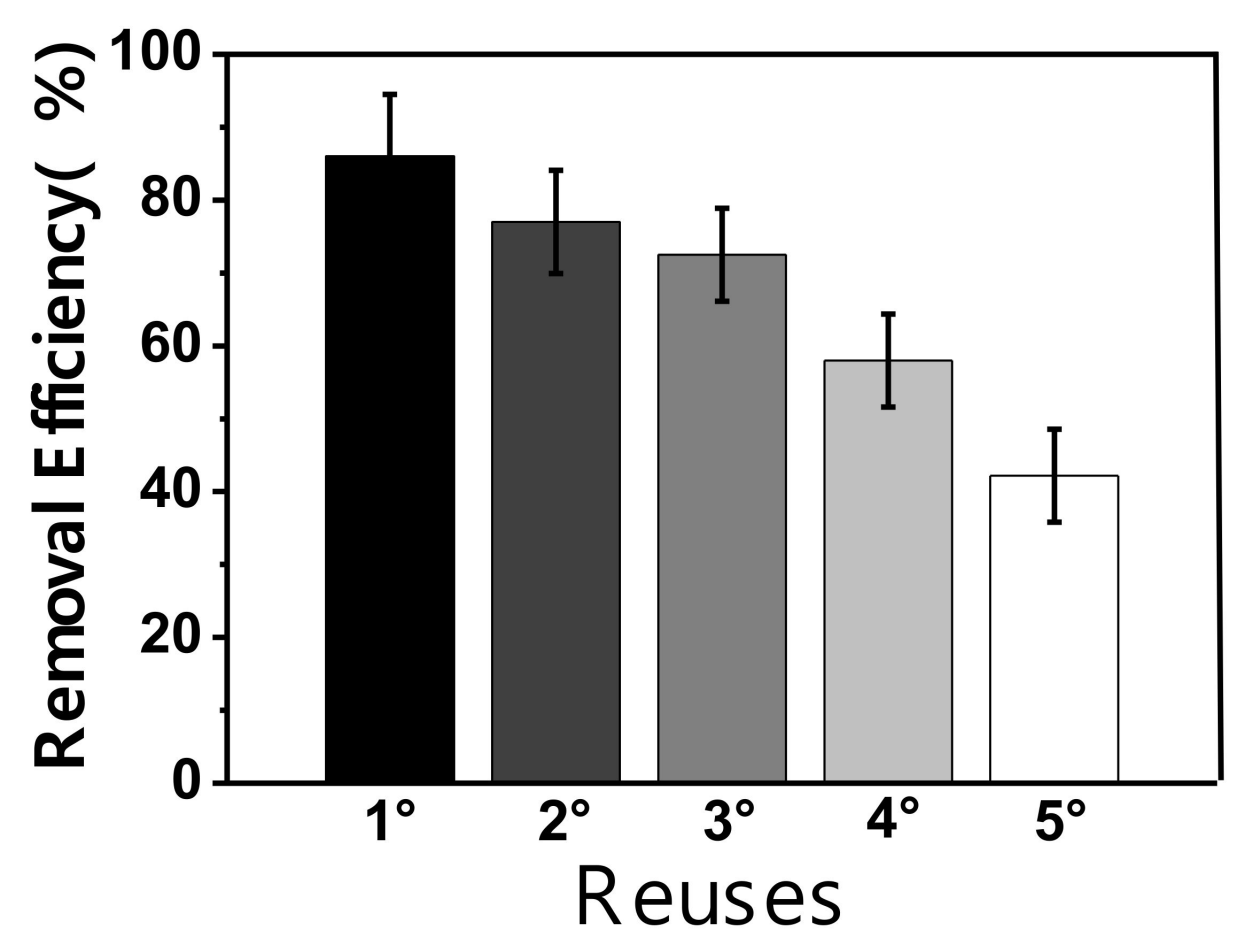
Reuse tests for methylene blue dye removal using the sample Fe_2_SiO_4_-Co_7_Fe_3_-SBA-15-700-2.0.

**Figure 8:**
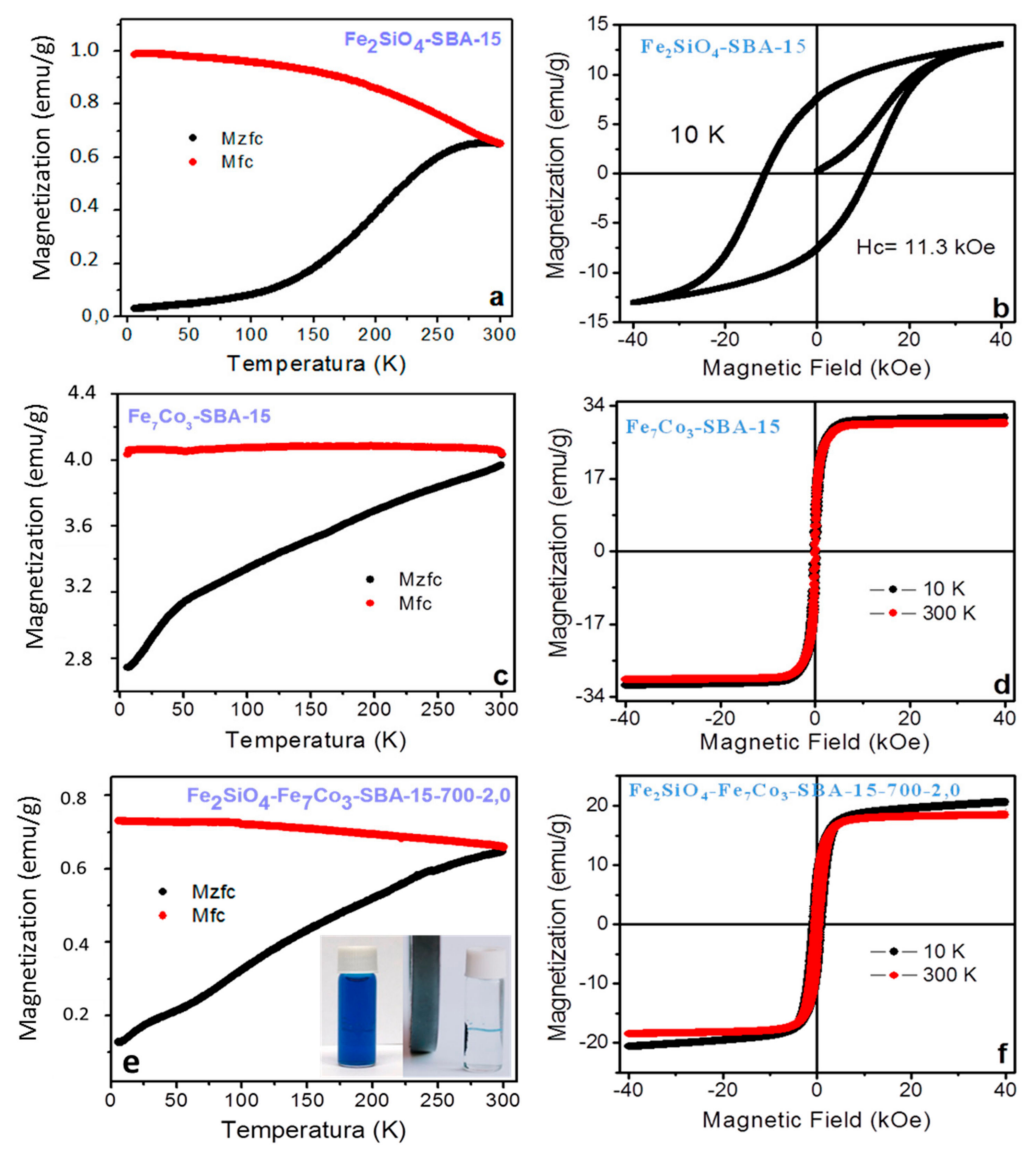
Magnetic properties for different samples. (**a**,**c**,**e**) magnetization zero field cooling (Mzfc) and magnetization field cooling (Mfc) magnetization curves; (**b**,**d**,**f**) Hysteresis cycles from vibrating sample magnetometer (VSM) analysis. The photo inserted in Figure 8f represents the nanocomposite separation by the application of a magnetic field after adsorption.

A reusability study was carried out for the material Fe_2_SiO_4_-Fe_7_Co_3_-SBA-15-700-2.0, Figure 7. The result related to adsorption capacity for the first test was 46 mg/g, for the second was 40 mg/g, the third was 37 mg/g, the fourth was 30 mg/g and, finally, for the fifth was 22 mg/g, indicating that there was a slight reduction in the adsorption capacity between recycle tests due to due to partial pore obstruction by methylene blue, however, the material was resistant since it has a little difference of 18 mg/g between the first and fifth reuse. It is important to highlight that this material is easily attracted by a magnet due to its magnetic properties, providing a simple way to separate the magnetic solid from the suspension. Figure 8 (insert) shows the solution before and after adsorption, illustrating the attraction of the nanocomposite by the application of an external magnetic field, which facilitates the recycle of this material.

### 2.8. Magnetic Properties (VSM, Mzfc, and Mfc)

The Figure 8a shows the measurements magnetization zero field cooling (Mzfc) and magnetization field cooling (Mfc) for nanocomposite Fe_2_SiO_4_-SBA-15. The Mzfc has a peak at 286 K and the Mfc show a large increase when the temperature decreases from 300 K to 6 K. The peak in the Mzfc may be related to a blocking temperature or to a spin glass transition. The M-H at 10 K shows a typical hysteresis loop characteristic of a hard magnetic material, Figure 8b. The maximum magnetic field of ± 40 kOe is not able to saturate magnetically the sample, i.e., there is a fraction of magnetic moments that are not aligned in the direction of the applied field. Thus, the ascending and descending M-H curves join at the field of ± 40 kOe. The coercivity field (Hc) and the remanent magnetization (Mr) are of 11.3 kOe and 7.5 emu/g, respectively. The magnetization at the field of 40 kOe is of *M* = 12.9 emu/g, and the ratio of the remanent magnetization and the magnetization at 40 kOe is of *Mr/M* = 0.58. The high coercivity field and high M/Mr values are typical of hard magnetic materials. In fact, for CoFe_2_O_4_ the value of Mr/Ms of 0.5 is typical of a system with uniaxial magnetocrystalline anisotropy and Mr/Ms > 0.5 is typical for a system with a cubic magnetocrystalline anisotropy (Ms is the saturation magnetization), therefore, the present sample seems to have a cubic magnetocrystalline anisotropy.

The Figure 8c shows the measurements Mzfc and Mfc for the solid Fe_7_Co_3_-SBA-15. The Mzfc does not show a peak and the Mfc data shows a quite small increase when the temperature decreases from 300 K to 6 K. The absence of a peak in the Mzfc curve indicates that the sample is thermally blocked below 300 K, i.e., the sample has a blocking temperature above 300 K. Figure 8d shows the M-H loops at 10 K and 300 K. The hystereses have a small coercivity field. Thus, they show typical behavior of soft magnetic materials, i.e., the sample has a low magnetocrystalline anisotropy. The hysteresis at 10 K and 300 K show coercivity fields of 390 Oe and 181 Oe, respectively. The Ms at 10 K and 300 K are of 31.2 and 29.9 emu/g, respectively. The Mr at 10 K and 300 K are of 12.2 and 8.3 emu/g, respectively, thus, their Mr/Ms values will be 0.39 and 0.28, respectively. In both curves the magnetization saturate quickly, beginning this process at a magnetic field of 10 kOe. These characteristics are typical of soft magnetic solids.

Taking into account that the Fe-Co metal nominal mass fraction is of 20 wt%, the Fe-Co-based phase corresponds to a 20 wt%. Therefore, the saturation magnetization for the Co_3_Fe_7_ phase will be 31.2/0.2 emu/g = 156 emu/g, this value is close to the expected value of 223 emu/g obtained for Co_3_Fe_7_ particles with a size of 180 nm [59]. The difference can be ascribed to surface effects due to the smaller particles size, i. e. the superficial spins are usually disordered and have a different magnetic behavior when compared with the spins at the core. The total surface area (per gram of sample) of very small particles is enhanced and the magnetic behavior of these nanoparticles is strongly affected by the superficial spins, leading to a smaller saturation magnetization.

For sample Fe_2_SiO_4_-Fe_7_Co_3_-SBA-15-700-2.0, the Mzfc does not show a peak in the range from 5 K to 300 K, indicating that the sample is in the thermal blocked regime, Figure 8e. The blocking temperature for this sample is above 300 K. The Mfc shows a typical behavior for blocked particles with low magnetocrystalline anisotropy. During the cooling under an applied magnetic field the spins are frozen in a particular direction depending on the strength of the applied magnetic field. The increase of Mzfc will be small with decrease in temperature if the sample has a low magnetocrystalline anisotropy. Figure 8f shows the M-H loops recorded at 10 K and 300 K. Both hysteresis show low coercivities fields of 940 Oe and 520 Oe, respectively. The magnetizations at 40 kOe were of 20.6 emu/g and 18.4 emu/g for the measurements recorded at 10 K and 300 K, respectively.

We have recorded an M-H measurement at 10 K after cooling the samples from 300 K under a magnetic field of 3 kOe, these measurements did not show a hysteresis shift due to an exchange bias effect. This result indicates that there is not a measurable magnetic coupling between the metal and the oxide phases. This coupling may occur at the interface between both phases and will shift the hysteresis to the left (right) when the coupling is ferromagnetic (antiferromagnetic). However, a chemical coupling may be present at the interface between the oxide and alloy and it will provide synergy between phases, which justifies the better adsorption capacity of the mixed phases compared to the same isolated phases (Figure 7). Table 3 shows the adsorption capacity of methylene blue from different adsorbents studied in the literature and compared with the present study [60,61,62,63,64,65]. It is possible to notice that the solid Fe_2_SiO_4_-Co_7_Fe_3_-SBA-15-700-2.0 which presented the best adsorption capacity has promising results compared to other solids containing Fe.

### 2.9. Mechanistic Proposal

Adsorption of environmental contaminants such as methylene blue on oxide-based surfaces and iron-containing metal alloys can occur through physical and chemical interactions between adsorbent and adsorbate. In physical adsorption, most adsorbed species occur in a very short time. However, a chemical bond between adsorbate and adsorbent requires longer contact time to reach equilibrium. Between the two stages, the adsorption rate is almost constant, since it has a large number of surface sites and vacancies available for adsorption during the initial stage, considering that all sites are free before adsorption. However, after certain interval of time, the remaining sites, present on the surface, are more difficult to be occupied, since most of the sites have been filled by the adsorbate [66]. Specifically, for methylene blue as adsorbate, due to its aromatic ring and N-H stretch in its structure, the main interactions studied are electrostatic, acid-base, ionic exchange, coordination, π-π interaction, and hydrogen bonds [66,67,68,69,70]. However, the interaction type is related to the intrinsic characteristics of each adsorbent [66].

**Scheme 1:**
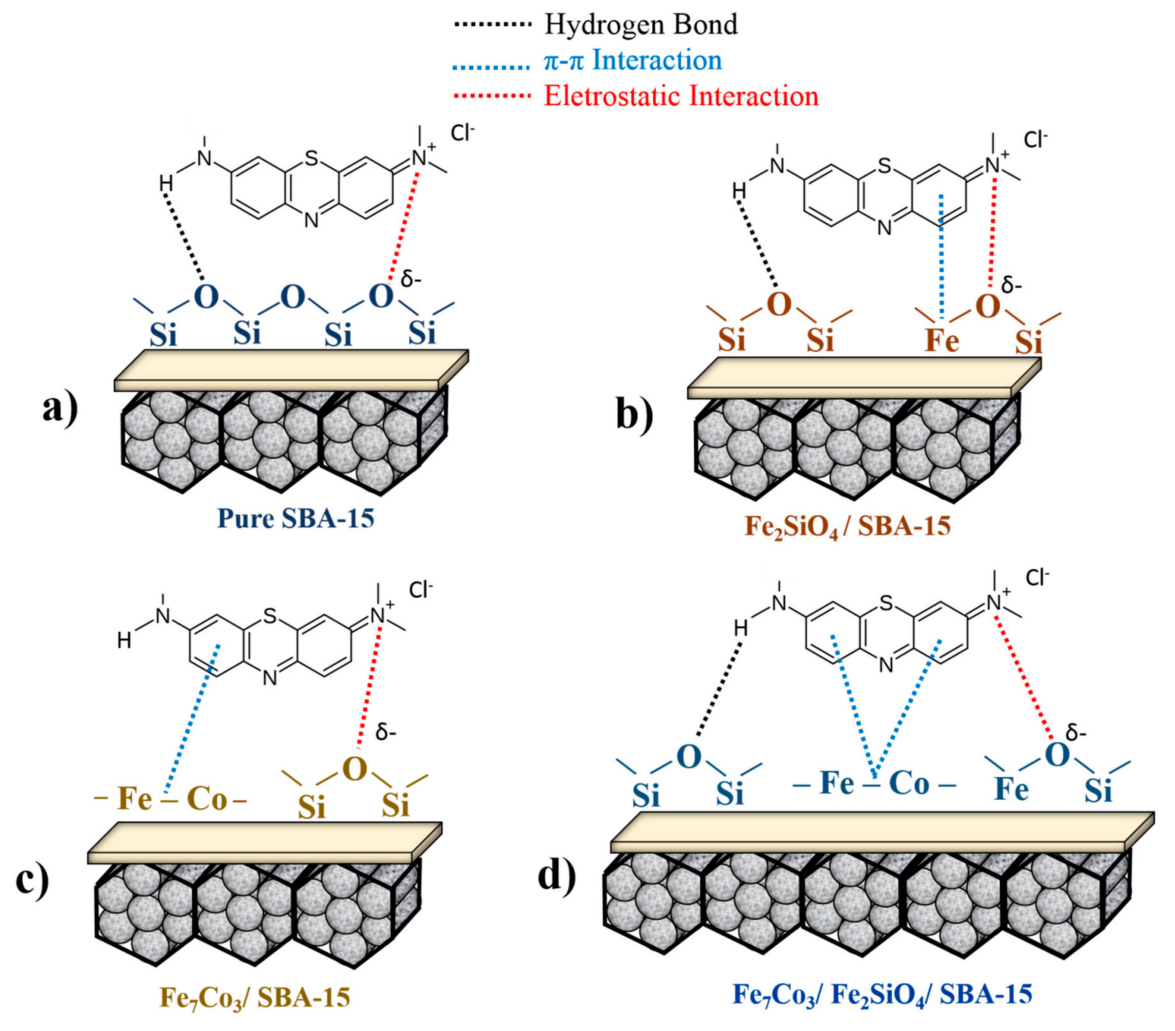
Mechanistic proposal for the methylene blue adsorption on the surface of different composites indicating the different types of interaction. (**a**) pure silica matrix; (**b**) oxide dispersed in SBA-15; (**c**) alloy supported in SBA-15; (**d**) oxide and alloy mixture dispersed in SBA-15.

Scheme 1 presents a proposal referring to the mechanism for methylene blue adsorption in the materials synthesized in the present study, indicating the main interactions between adsorbent and adsorbate. In Scheme 1a, the test with pure SBA-15 demonstrated low adsorption capacity (Figure 6a), however, it is possible to predict interactions of the lattice oxygen present in the silica based matrix with N present in the methylene blue structure. In addition, it is also possible to have interactions of nitrogen bonded with H in the dye structure with the lattice oxygens present with amorphous silica through stronger hydrogen binding interactions.

In the case of Scheme 1b, the presence of Fe_2_SiO_4_ oxide dispersed on SBA-15 provides a significant gain in methylene blue adsorption, considering that the number of possibilities for interactions is greater. In this case, the structure has more lattice oxygen sites present in the structure of silica and iron-cobalt oxides. Moreover, the presence of Fe causes π-π interactions with aromatic rings from the dye, justifying the higher adsorption capacity for the Fe_2_SiO_4_-SBA-15 sample when compared to pure SBA-15 (Figure 6a).

Scheme 1c displays the material containing the pure Fe_7_Co_3_ alloy supported on SBA-15. The result was more significant when compared to the two schemes previously described. In the case of pure alloy dispersed in silica matrix, the presence of metallic Fe and Co intensifies π-π interactions with the aromatic rings of dye structure compared to Fe^2+^/Fe^3+^ from oxide, justifying the higher adsorption capacity of the Fe_7_Co_3_ -SBA-15 solid compared to the sample Fe_2_SiO_4_-SBA-15.

Finally, Scheme 1d presents the composite containing the mixture of alloy and oxide-based phases supported on SBA-15. In this case, it is possible to propose the existence of three types of interactions (electrostatic, π-π, and hydrogen bonding). Fe and Co based metals present in oxide and alloy structure have the adsorption ability through π-π interaction with aromatic rings of dye. Lattice oxygens present in the structure of silica and fayalite can also adsorb dye molecules through electrostatic interaction or hydrogen binding. The higher adsorption capacity due to the presence of the mixture of phases, Fe_7_Co_3_ and Fe_2_SiO_4_, when compared to the same isolated phases, may be related to the synergistic effect between the fayalite and alloy phases. Electron transfer from π-π interactions is favored due to the synergistic effects of Fe^0^ and Fe^2+^, justifying the higher adsorption capacity for the Fe_2_SiO_4_-Fe_7_Co_3_-SBA-15-700-2.0 sample compared to Fe_7_Co_3_-SBA-15 and Fe_2_SiO_4_-SBA-15 solids. It is important to highlight that previous studies have already mentioned the positive effect of the Fe^0^-Fe^2+^-Fe^3+^ mixture due to the synergistic effects on dyes adsorption, corroborating with the results obtained in the present study [58,70].

## 3. Materials and Methods

### 3.1. Synthesis of SBA-15 Silica

The mesoporous silica matrix was prepared by the hydrothermal method previously described in the literature using as starting reagents the triblock copolymer P123 (EO_20_PO_70_EO_20_), hydrochloric acid 37% *m*/*v*, distilled water, and tetraethylosilicate (TEOS) as a silica source [39]. The synthesis was performed to obtain 10 g of final material. Initially, it was dissolved 16.5 g of the Pluronic P123 copolymer into 519.2 g of distilled water and 96.7 g of hydrochloric acid. The solution was kept under constant magnetic stirring for 3 h at 35 °C. Then, after the complete dissolution of the P123 copolymer, the formed gel was transferred to a teflon autoclave and submitted to heat treatment in a drying oven at 100 °C during 24 h. The obtained solid was removed by filtration using distilled water and vacuum pump, and a pH = 6 was obtained. Finally, the dry material was calcined at 600 °C for 6 h with a heating rate of 2 °C/min. The solid containing only the silica matrix was named as pure SBA-15.

### 3.2. Synthesis of Dispersed Oxide in SBA-15 Using the Incipient Impregnation Method

The synthesis of the composite was performed by the incipient impregnation method. The reagents were weighed in order to obtain 20% by weight of dispersed oxide in the silica matrix with a Fe and Co molar ratio of 2:1. Initially, two distinct solutions were prepared, the first solution consisting of 0.861 g of iron nitrate (III) nonahydrate {Fe(NO_3_)_3_.9H_2_O – 404 g/mol} and a second solution containing 0.310 g of cobalt nitrate (II) hexa-hydrated {Co(NO_3_)_2_.6H_2_O – 290.7 g/mol}. Both were diluted in distilled water. Then, both solutions were mixed into a single flask and stirred until a uniform solution was obtained. Subsequently, this solution containing cobalt and iron ions, was slowly transferred (using a Pasteur pipette) to the support (SBA-15) until it was observed that the wet point was reached. Subsequently, the material was dried in a drying oven at 100 °C during 1 h for evaporation of solvents. The process was repeated until the solution containing Fe^3+^ and Co^2+^ ions was completely impregnated in the silica matrix. Finally, the material was calcined at 700 °C with heating rate of 5 °C/min during 2 h. The final oxide obtained was designated as Fe_2_SiO_4_-SBA-15.

### 3.3. Synthesis of the Composite Fe_2_SiO_4_-Fe_7_Co_3_ Dispersed in the Mesoporous SBA-15

The synthesis of the oxide-alloy mixture was performed using a fixed-bed quartz microreactor with 50 mg of dispersed oxide in the SBA-15 previously described (Fe_2_SiO_4_-SBA-15 sample). The temperature and the percentage of the reducing gas were varied in order to observe the effect of these parameters on the formation of the oxide-alloy mixture, considering that the mixture was formed by the reduction of oxide to alloy.

Initially, a pretreatment was performed under nitrogen flow rate (30 mL/min) for 30 min at 350 °C in order to remove the water and CO_2_ physisorbed. Then, two studies were conducted, the first study varying the reaction temperature with heating rate of 13 °C/min and with the percentage of hydrogen (both constants), using 2% of H_2_ and 98% of N_2_ with total flow of 25 mL/min at four distinct temperatures 690, 700, 710, and 720 °C, respectively. The second study varying the ratio of H_2_/N_2_, i.e., the percentage of H_2_ in the mixture, and keeping the temperature at 700 °C as well as the heating rate and total flow rate. The different percentages of H_2_ in the H_2_/N_2_ mixture were of 0.5, 1.0, 1.5, and 2.0 % of H_2_. The Fe_2_SiO_4_-Fe_7_Co_3_ phase mixtures (oxide-alloy) were named according to the temperature and percentage of H_2_ used for each sample. The different solids were named as Fe_2_SiO_4_-Fe_7_Co_3_-SBA-15-X-Y, where X represents the reduction temperature and Y the percentage of H_2_. The different samples prepared are described in Table S5, according to the reduction temperature and the amount of H_2_ used. For comparison, a sample containing pure alloy dispersed in the SBA-15 mesoporous was synthesized. This sample was synthesized using pure H_2_ with a total flow rate of 25 mL/min, at the temperature of 900 °C using a heating rate of 13 °C/min. The pure alloy-containing solid was named Fe_7_Co_3_-SBA-15.

### 3.4. Adsorption of Dyes Using Fe_2_SiO_4_-Fe_7_Co_3_ Composite Dispersed in SBA-15

Solutions of different dyes (0.05 g/L) methylene blue (MB), methyl orange (MO), and rodamine B (RB) were prepared by dissolving 0.0125 g of dye into 250 mL of distilled water. In a typical adsorption experiment, 20 mg of the synthesized adsorbents were suspended in 20 mL of dye solution. The resulting mixture was kept under mechanical stirring, 200 rpm, for 12 h at room temperature. The amount of adsorbed dyes was accompanied by the standard UV/VIS absorption method. Magnetic separation of the nanocomposite was performed before the analysis to avoid possible interference of suspended particles in UV/VIS analysis [71]. Specifically, for the sample Fe_2_SiO_4_-Fe_7_Co_3_-SBA-15-700-2.0, reusability tests were performed to evaluate the recycle capacity of the nanocomposite.

### 3.5. Characterization of Nanocomposite Fe_2_SiO_4_-Fe_7_Co_3_-SBA-15

The X-ray diffraction analyses were performed on a Bruker D2 Phaser diffractometer (Atibaia, SP, Brazil) using CuKα radiation (*λ* = 1.54Å) with a Ni filter, with a 0.02° step, 10 mA current, and a 30 kV voltage using a detector Lynxeye to determine the crystalline structures of the synthesized solids. The analysis was performed with a 2θ angle between 10 and 90 degrees. Phase identification was performed using the X-Pert HighScore Panalytical software Anto Amaro, SP, Brazil and the 2003 JCPDS-ICDD database [72,73]. Rietveld refinements were done using GSAS software [74] and EXPGUI interface [75], after determining instrumental broadening by means of refining a LaB6 NIST standard sample. The modified Pseudo–Voigt function (Thompson–Cox–Hastings) was chosen to adjust the profiles of the diffraction peaks for the identified crystalline phases. The full width at half height (FWHM) of the peaks was used to calculate the crystallite size using the Scherrer equation [76]. From the refinement it was possible to obtain the percent phase between alloy and oxide for each experimental.

For temperature-programmed reduction (TPR) analyses, two different reduction flow ratios were used in order to confirm the effect of H_2_ content on the formation of Fe_7_Co_3_ alloy and Fe_2_SiO_4_-Fe_7_Co_3_ based composite. Initially, a pre-treatment was performed, where the samples were pretreated for 45 min at 120 °C under N_2_ atmosphere in order to eliminate possible physisorbed impurities. The analyses occurred at two different H_2_/N_2_ ratios, flow rate of 25 mL/min for the total mixture, and heating rate of 13 °C/min from room temperature to 950 °C. A thermal conductivity detector (TCD) was used to monitor H_2_ consumption and determine the complete reduction temperature of iron and cobalt oxides to alloy.

The N_2_ physisorption analysis were performed at a temperature of 77 K (−196 °C) in a gas adsorption analyzer (Atlanta, USA) ASAP 2020 Physisorption/Micromeritics model in order to obtain the textural properties of the catalysts. Prior to analysis, the solids were degassed under vacuum at 200 °C for 2 h. From the obtained isotherms were determined the specific surface area from the BET method, pore volume, and pore diameter values.

The morphology of the SBA-15 silica support and the dispersion of iron and cobalt were investigated by high resolution scanning electron microscopy (SEM-FEG) with a Hitachi TM-3000 microscope. The accelerating voltage was 5–15 kV and it was applied different magnifications (15 a 30,000×), Chiyoda, Tokyo, JP. The histogram of the particle size distribution was extracted using the Image-J (Bethesda, Maryland, USA) program taking into account approximately 135 particles.

Confirmation of the morphology for the SBA-15 silica matrix and the particle size distribution were also investigated by transmission electron microscopy (TEM) with an acceleration voltage of 120 kV (Jeol, JEM-2100, with EDS, Thermo scientific, Waltham, MA, USA). The nanocomposites were prepared by placing one drop of dispersion on a carbon coated copper grid (300 mesh). The histogram of the particle size distribution was also extracted from the images obtained using the Image-J software taking into account approximately 80 particles.

^57^Fe Mössbauer spectra at 300 K were recorded in the transmission mode using a spectrometer (SEECo, Minneapolis, MN, USA) with triangular velocity sweep. The 14.4 keV γ-radiation source is ^57^Co in a Rh matrix with an activity of 20 mCi.

The magnetic characterization was performed with a Dynacool-Physical Property Measurement System-PPMS equipped with a vibrating sample magnetometer (Quantum Design, California, CA, USA). The zero field-cooled (Mzfc) and field-cooled (Mfc) magnetizations measurements are recorded in the temperature range between 5 K and 300 K, under a magnetic field of 80 Oe. The isothermal magnetization, M-H curves, are recorded at 10 K and 300 K with a maximum magnetic field of ± 40 kOe. In this case, the sample is cooled in absence of applied magnetic field.

The XPS analyses were performed on a Kratos Ultra DLD, Kyoto, JP. spectrometer using monochromated Al K radiation (10 mA, 15 kV). All spectra were taken in the hybrid mode (combined electrostatic and magnetic lens). All spectra were referenced to the C1s line at binding energy 284.6 eV, characteristic of everpresent adventitious carbon (C-C and C-H). This peak position was obtained after Shirley background subtraction and decomposition of the C1s peak envelope using a Gaussian-Lorentzien (70%–30%) curve fit. All other photoelectron peaks were background-subtracted and fitted in the same manner. Quantification was performed using the VISION software supplied. The relative sensitivity factors (RSF) applied are inherent to this software and incorporate Wagner photoelectron cross-sections and analyzer transmission correction.

## 4. Conclusions

Different nanocomposites based on iron and cobalt dispersed on SBA-15 were successfully synthesized and applied in the adsorption of methylene blue dye. The studied parameters, reduction temperature and H_2_ content in the total flow rate, directly influence the amount of the formed phases, the physicochemical properties of the nanocomposite and consequently on the adsorption capacity. The increase in reduction temperature and amount of H_2_ contributed significantly to the formation of the alloy phase (Fe_7_Co_3_) from the metal oxide. The synthesis method used led to the formation of nanoparticles indicating a high dispersion of the iron-cobalt phases in mesoporous silica. The characteristics of the silica matrix were slightly altered after the impregnation and formation of the nanocomposite due to partial pores filling, however, the mesoporosity characteristic of the SBA-15 was maintained for the different nanocomposites. Adsorption tests confirmed that the material using 2% of hydrogen for total flow rate and reduction temperature of 700 °C presented the highest adsorption capacity for methylene blue dye. The nanocomposite containing the mixture of alloy and oxide supported on SBA-15 has higher adsorption capacity compared to the same isolated phases due to the synergistic effect between the fayalite and Fe_7_Co_3_ alloy phases. The obtained nanocomposites presented better performance for methylene blue adsorption compared to other dyes (methyl orange and rhodamine B) and can be easily separated from dispersion by simple application of an external magnetic field, facilitating its recycle. Reusability tests revealed a drop of 44% in adsorption capacity comparing the first with the fifth test.

## Figures and Tables

**Table 1:** Rietveld refinement results for solids at different reduction temperatures and the different amounts of H_2_.

Sample	Phase Percentage (%)		Crystallite Size (nm)	
	Fe_7_Co_3_	Fe_2_SiO_4_	Fe_7_Co_3_ (44°) **	Fe_2_SiO_4_ (35°) **
Fe_2_SiO_4_-Fe_7_Co_3_-SBA-15-690-2.0 *	23	71	17	12
Fe_2_SiO_4_-Fe_7_Co_3_-SBA-15-700-2.0	42	58	16	15
Fe_2_SiO_4_-Fe_7_Co_3_-SBA-15-710-2.0	44	56	15	16
Fe_2_SiO_4_-Fe_7_Co_3_-SBA-15-720-2.0	77	23	14	16
Fe_2_SiO_4_-Fe_7_Co_3_-SBA-15-700-0.5	15	85	16	14
Fe_2_SiO_4_-Fe_7_Co_3_-SBA-15-700-1.0	21	79	14	10
Fe_2_SiO_4_-Fe_7_Co_3_-SBA-15-700-1.5	27	73	20	19

* For the sample with the lowest reduction temperature, 6% of impurity was observed referring to the SiO_2_ phase. ** Reflection used to calculate the crystallite size using the Scherrer equation.

**Table 2:** Textural properties for the different samples varying reduction temperature and H_2_ content.

Sample	S_BET_ [m²/g]	V_MP_ [cm³/g]	V_MPP_ [cm³/g]	V_TP_ [cm³/g] P/P_0_ = 0.98
SBA-15	640	0.03	0.81	0.95
Fe_2_SiO_4_-SBA-15	510	0.01	0.56	0.69
Fe_7_Co_3_-SBA-15	420	0.01	0.54	0.67
Fe_2_SiO_4_-Fe_7_Co_3_-SBA-15-690-2.0	430	0.01	0.52	0.65
Fe_2_SiO_4_-Fe_7_Co_3_-SBA-15-720-2.0	460	0.01	0.55	0.68
Fe_2_SiO_4_-Fe_7_Co_3_-SBA-15-700-0.5	435	0.01	0.52	0.64
Fe_2_SiO_4_-Fe_7_Co_3_-SBA-15-700-2.0	435	0.01	0.54	0.66

**Table 3:** Comparison of the adsorption capacity with other previously published papers using magnetic materials for methylene blue dye.

Material	q (mg/g)	Reference
Fe_2_SiO_4_-Co_7_Fe_3_-SBA-15-700-2.0	49	Present study
Zinc-doped cobalt ferrite	27.79	[60]
MnFe_2_O_4_	20.7	[61]
Magnetic carboxyl functional nanoporous	57.74	[62]
Magnetic MWCNT nanocomposite	15.74	[63]
FeFe_2_O_4_	42.35	[64]
ZnO/ZnFe_2_O_4_	32.27	[65]

## Supplementary Materials

The following are available online at https://www.mdpi.com/1420-3049/25/4/1016/s1.
