# Supplementary material for: Synthesis of Fe2SiO4-Fe7Co3 Nanocomposite Dispersed in the Mesoporous SBA-15: Application as Magnetically Separable Adsorbent
Source: Molecules. 2020 Feb 24;25(4):1016. doi: 10.3390/molecules25041016 (PMC7071072; doi:10.3390/molecules25041016)
Supplement: Supplementary file 1 [file molecules-25-01016-s001.pdf]

Supplementary Material

Synthesis of Fe_2SiO_4-Fe_7Co_3 Nanocomposite Dispersed in the Mesoporous SBA-15: Application as Magnetically Separable Adsorbent

Monickarla Teixeira Pegado da Silva [1], Felipe Fernandes Barbosa [1], Marco Antonio Morales Torre [2], Jhonny Villarroel-Rocha [3], Karim Sapag [3], Sibele B.C. Pergher [1,*] and Tiago Pinheiro Braga [1,*]

[1] Laboratório de Peneiras Moleculares, Instituto de Química, Universidade Federal do Rio Grande do Norte, 59078-970 Natal, RN, Brazil; monickarla@yahoo.com.br (M.T.P.d.S.); felipefbarboza@outlook.com (F.F.B.)

[2] Departamento de Física, Universidade Federal do Rio Grande do Norte, 59078-970 Natal, RN, Brazil; morales@dfte.ufrn.br

[3] Laboratorio de Sólidos Porosos, Universidad Nacional de San Luis, Instituto de Física Aplicada, 5700, D5700BPB San Luis, Argentina; jhoviro@gmail.com (J.V.-R.); ksapag@gmail.com (K.S.)

* Correspondence: tiagoquimicaufrn@gmail.com (T.P.B.); sibelepergher@gmail.com (S.B.C.P); Tel: +55-84-33422323 (T.P.B.)

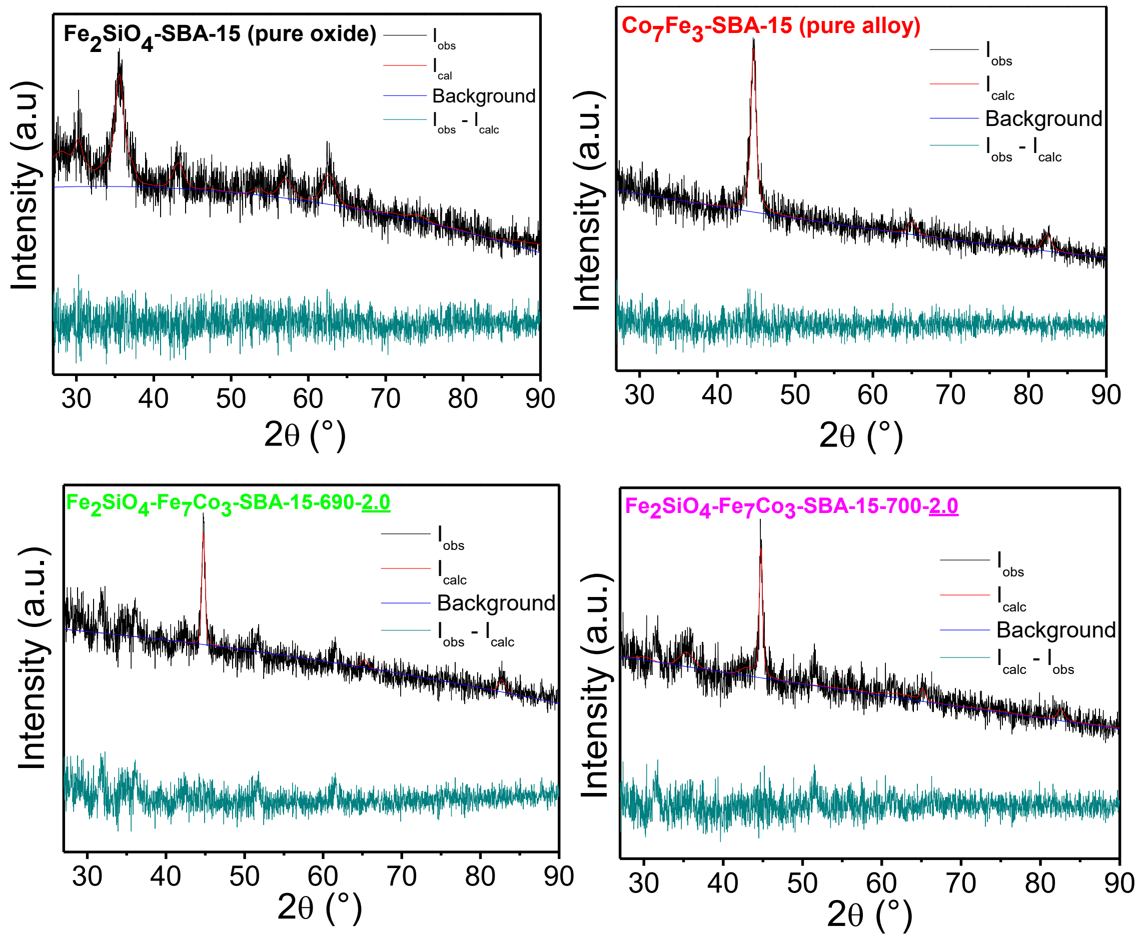

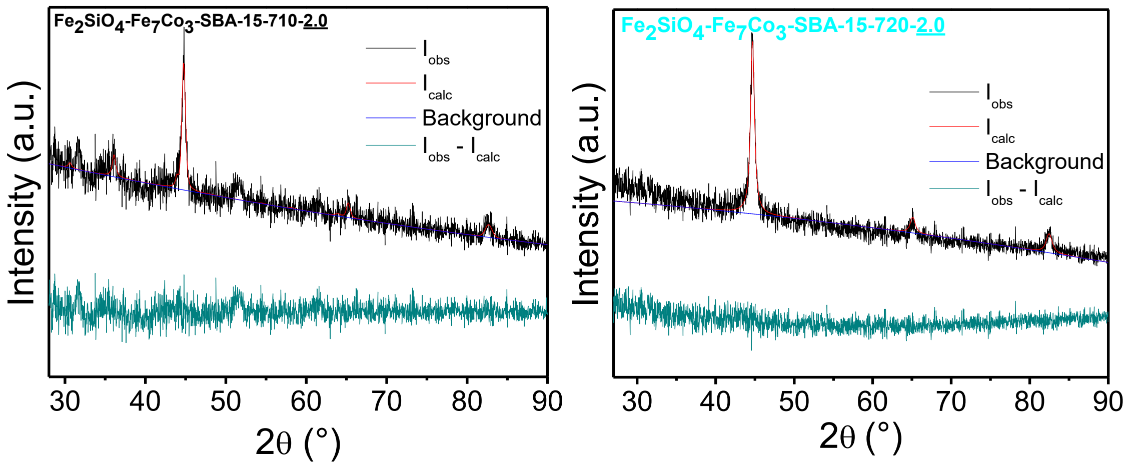

Figure S1. Results obtained by the Rietveld refinement for the study concerning the reduction temperature variation, for the oxide dispersed on SBA-15 and for the alloy dispersed on SBA-15.

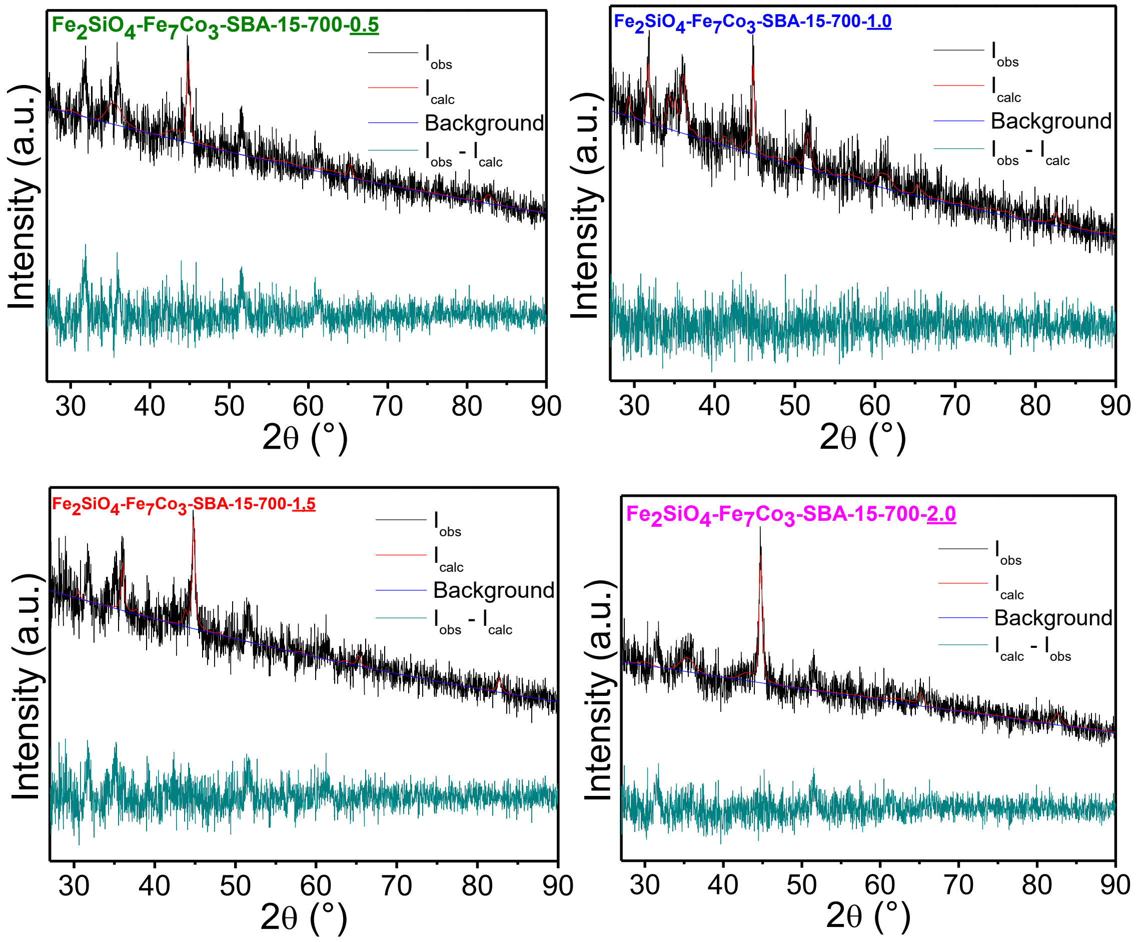

Figure S2. Results obtained by Rietveld refinement for the study related to hydrogen content variation.

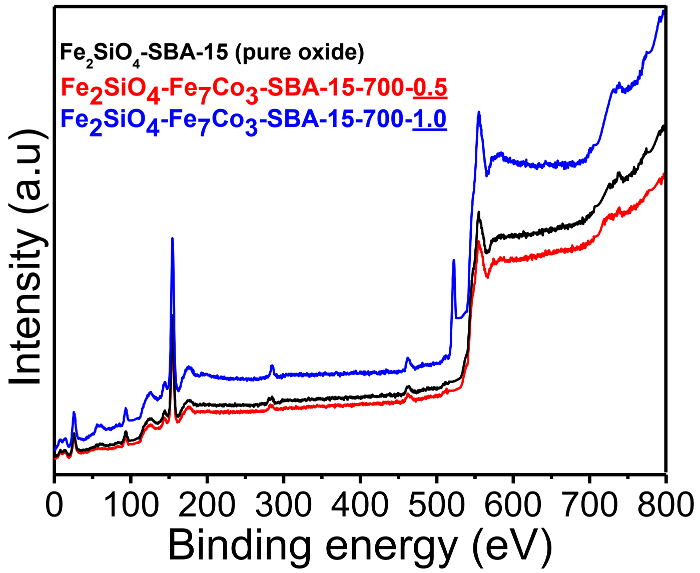

Figure S3. XPS wide scan spectrum for solids containing oxide dispersed on SBA-15; and the mixture oxide and alloy dispersed on SBA-15.

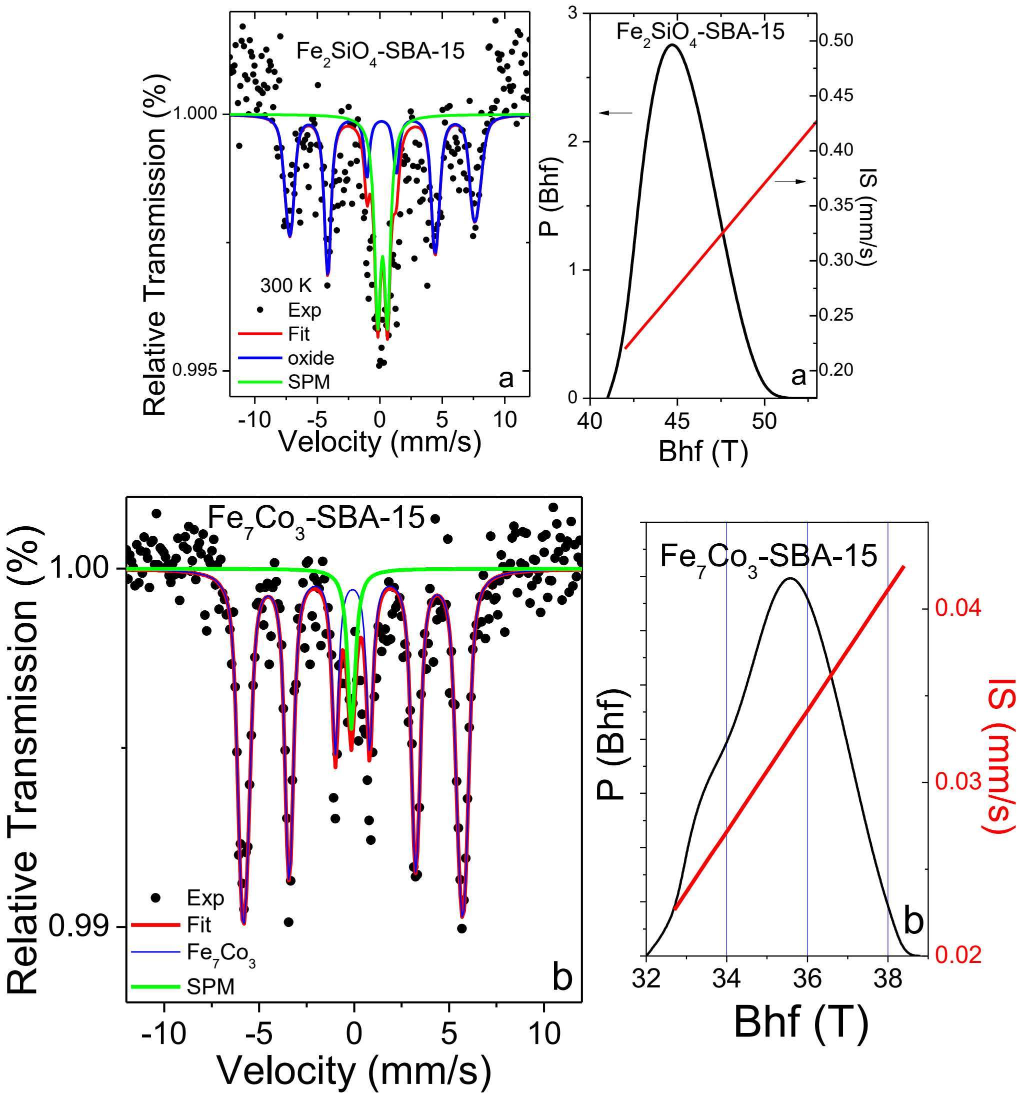

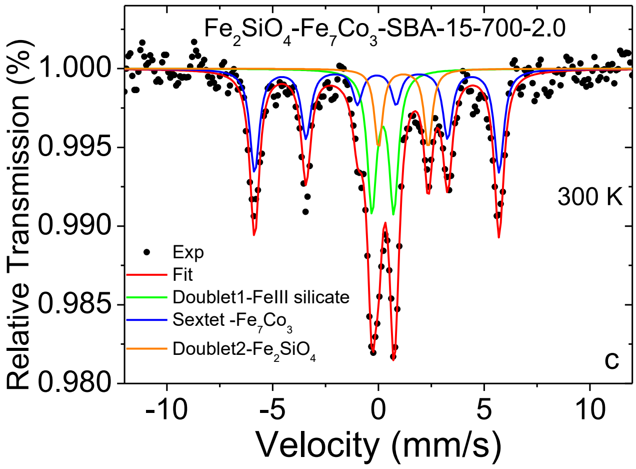

Figure S4. Mössbauer spectrum at 300K. (a) for the sample Fe_2SiO_4-SBA-15; (b) for the material Fe_7Co_3-SBA-15; and (c) for the solid Fe_2SiO_4-Fe_7Co_3-SBA-15-2.0.

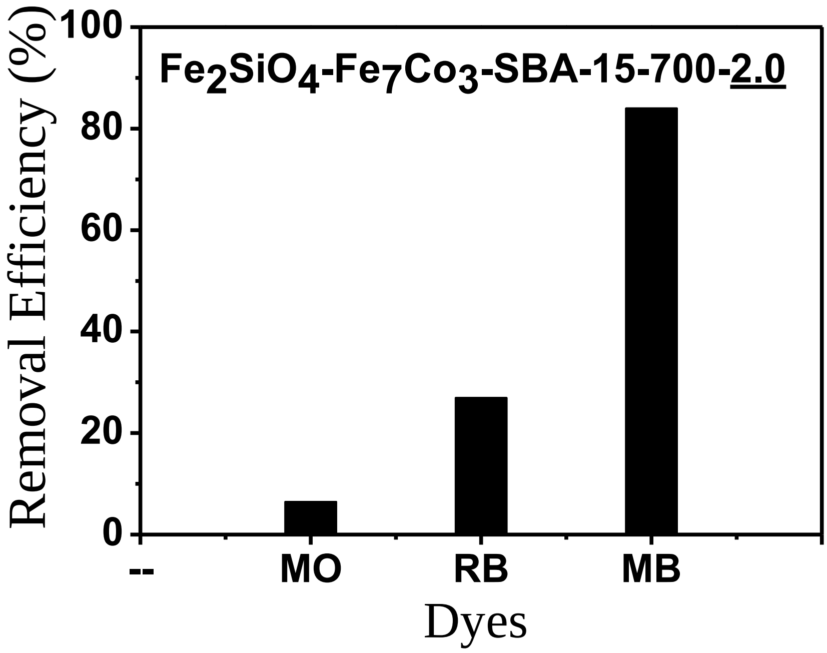

Figure S5. Degradation performance of methylene blue (MB), methyl orange (MO) and rhodamine B (RB) for the nanocomposite Fe_2SiO_4-Fe_7Co_3-SBA-15-2.0.

Table S1. Different synthesized samples according to reduction temperature and percentage of H_2.

Samples	Reduction temperature	Percentage of H_2
Fe_2SiO_4-Fe_7Co_3-SBA-15-690-2.0	690 °C	2.0%H_2 / 99.5%N_2
Fe_2SiO_4-Fe_7Co_3-SBA-15-700-2.0	700 °C	2.0%H_2 / 99.0%N_2
Fe_2SiO_4-Fe_7Co_3-SBA-15-710-2.0	710 °C	2.0%H_2 / 98.0%N_2
Fe_2SiO_4-Fe_7Co_3-SBA-15-720-2.0	720 °C	2.0%H_2 / 98.0%N_2
Fe_2SiO_4-Fe_7Co_3-SBA-15-700-0.5	700 °C	0.5%H_2 / 99.5%N_2
Fe_2SiO_4-Fe_7Co_3-SBA-15-700-1.0	700 °C	1.0%H_2 / 98.5%N_2
Fe_2SiO_4-Fe_7Co_3-SBA-15-700-1.5	700 °C	1.5%H_2 / 98.0%N_2
Fe_2SiO_4-Fe_7Co_3-SBA-15-700-2.0	700 °C	2.0%H_2 / 99.0%N_2

Table S2. Hyperfine parameters from Mössbauer spectra for iron-based samples.

Sample	Spectrum	IS (mm/s)	QS (mm/s)	B_{hf} (T)	Area (%)
	Sextet	0.025	0.015	35.8	52
Fe_2SiO_4-Fe_7Co_3-SBA-15-700-2.0	Doublet 1	0.311	1.033	0	34
	Doublet 2	1.179	2.349	0	14
Fe_7Co_3-SBA-15	Sextet	<0.032>	0.024	<35.7>	92
	SPM	0.031	-	-	8
Fe_2SiO_4-SBA-15	Fe-oxide	<0.28>	0.070	<46.2>	65
	SPM	0.31	0.78	-	35

Table S3. Wall thickness for SBA-15 before and after impregnation extracted from low angle XRD data and N_2 isotherms.

**Sample**	**d_{100} / (nm)**	**a_0 / (nm)**	**D_P / (nm)**	**W_t / (nm)**
SBA-15	9.7	11.2	7.4	3.8
Fe_7Co_3-SBA-15	9.6	11.1	6.8	4.3

a_0: lattice parameter= $2d_{100}/\sqrt{3}$; W_t: wall thickness = $a_0 - D_P$; D_P: Average pore diameters obtained from N_2 isotherms.
